# Robust Distance Correlation for Variable Screening

**DOI:** 10.1002/sta4.70094

**Published:** 2025-08-22

**Authors:** Tianzhou Ma, Fan Yang, Hongjie Ke, Zhao Ren

**Affiliations:** 1 Department of Epidemiology and Biostatistics, University of Maryland, College Park, Maryland, USA; 2 Department of Statistics, University of Pittsburgh, Pittsburgh, Pennsylvania, USA

**Keywords:** distance correlation, false discovery rate, Huber loss, robustness, sure screening property, variable selection

## Abstract

In modern statistical applications, identifying critical features in high-dimensional data is essential for scientific discoveries. Traditional best subset selection methods face computational challenges, while regularization approaches such as Lasso, SCAD and their variants often exhibit poor performance with ultrahigh-dimensional data. Sure screening methods, widely used for dimensionality reduction, have been developed as popular alternatives, but few target heavy-tailed characteristics in modern big data. This paper introduces a new sure screening method, based on robust distance correlation (‘RDC’), designed for heavy-tailed data. The proposed method inherits the benefits of the original model-free distance correlation-based screening while robustly estimating distance correlation in the presence of heavy-tailed data. We further develop an FDR control procedure by incorporating the Reflection via Data Splitting (REDS) method. Extensive simulations demonstrate the method’s advantage over existing screening procedures under different scenarios of heavy-tailedness. Its application to high-dimensional heavy-tailed RNA-seq data from The Cancer Genome Atlas (TCGA) pancreatic cancer cohort showcases superior performance in identifying biologically meaningful genes predictive of MAPK1 protein expression critical to pancreatic cancer.

## 1 | Introduction

Data with a much larger number of features than sample size are frequently seen in modern statistical applications, ranging from genomic research, biomedical imaging to signal processing problems. High-dimensional variable selection has become fundamentally important to many scientific discoveries nowadays. For example, oncologists apply variable selection methods to identify genes most predictive of cancer prognosis from tens of thousands of candidate genes. Classical variable selection methods such as the best subset selection using AIC or BIC criterion suffer from an NP-hard combinatorial problem, and the computation becomes unaffordable with a large number of features. Since the late 1990s, various regularization methods have been developed and extensively applied for feature selection in high-dimensional regression and classification models. These methods include but are not limited to Lasso (Tibshirani 1996), SCAD (Fan and Li 2001), elastic net (Zou and Hastie 2005), the adaptive Lasso (Zou 2006) and MCP (Zhang 2010). One can refer to Fan and Lv (2010) for a detailed overview of variable selection methods in high-dimensional models. As the number of features p grows exponentially with the sample size n in an ultrahigh-dimensional problem, however, many regularization methods perform poorly owing to the low computational efficiency and unstable algorithm. In a seminal work, Fan and Lv (2008) proposed a sure independence screening (SIS) method by first ranking and selecting features based on their marginal correlations with the response in a linear model before applying any regularization methods. SIS is appealing in practice due to its ability to rapidly reduce the dimension in the original model while keeping the most important features in the reduced set with large probability, a characteristic known as ‘sure screening property’. From then on, variable screening has drawn considerable attention, and a wide variety of screening methods have been developed for different models or problems in the past decade. Fan et al. (2009) and Fan and Song (2010) developed a more general version of independent screening by ranking the maximum marginal likelihood estimators for the generalized linear model. Other papers have proposed sure screening methods for nonparametric or semi-parametric regression (Fan et al. 2011; Liu et al. 2014; Chang et al. 2016), for linear model with interaction terms (Niu et al. 2018), for quantile regression (He et al. 2013; Wu and Yin 2015; Ma et al. 2017), for censoring data (Zhao and Li 2012; Gorst-Rasmussen and Scheike 2013; Song et al. 2014), for Gaussian graphical model (Liang et al. 2015), for classification problems (Mai and Zou 2012; Pan et al. 2016; Xie et al. 2020) and among others. In addition to those methods targeting a class of specific models, Zhu et al. (2011), Li et al. (2012) and Mai and Zou (2015) proposed model-free screening procedures for a more general usage when the model structure cannot be clearly specified.

A majority of regularization methods and marginal correlation-based screening methods assumed light-tailed distribution for response and covariates (Fan and Lv 2008; 2010). The violation of such an assumption is common in practice. Non-Gaussian and heavy-tailed distribution is a stylized feature of high-dimensional data such as microarray, next-generation sequencing data in genomics and neuroimaging data. For example, the gene expression measured by either bulk RNA-seq or single-cell RNA-seq techniques displays both zero inflation and heavy-tail behavior that approximate a power law (Townes and Irizarry 2020). To tackle such a problem, numerous robust regularization methods have been developed in the literature based on quantile regression or least absolute regression (Li and Zhu 2008; Wu and Liu 2009; Fan et al. 2014). However, these methods are essentially targeting median estimation instead of mean estimation. Another line of work, as in Zhong et al. (2016); Chen et al. (2018); Mai et al. (2023), applies robust, rank-based transformations and computes utility measures for variable screening based on the transformed data. It is worth noting, however, that such rank-based transformations may alter the relative ranking of predictors under the original utility measure. In a deviation analysis, Catoni (2012) showed that the empirical mean deviates significantly in the presence of heavy-tailed errors. By revisiting the classical Huber loss as a way of robustification (Huber 1964), Fan et al. (2017) and Sun et al. (2020) proposed Huber-type estimators of regression coefficients and derived their nonasymptotic concentration results in a linear model under mild moment conditions on the error. In these methods, a tuning parameter, called robustification parameter, needs to be specified beforehand to balance between robustness and bias of estimation. A similar Huber estimator has been proposed for estimating the high-dimensional covariance matrix with the robustification parameter determined via empirical cross-validation (Avella-Medina et al. 2018) or in a data-driven fashion (Ke et al. 2019). However, few methods have been developed for the robust estimation of the original measures (e.g., Pearson correlation or distance correlation) directly used in variable screening when heavy-tailed distributions are observed.

First introduced by Székely et al. (2007) and then used as a utility for variable screening in Li et al. (2012), distance correlation (DC) is a new measure of dependence and has impressive properties that make it useful in feature screening and stand out among all screening procedures. Specifically, distance correlation is robust to model misspecification and equivalent to Pearson correlation in screening when the normality assumption holds. In addition, it measures the dependence between two random vectors so it can be readily used for multivariate responses or group-wise predictors often seen in real data. Later work on DC considers bias correction in DC estimation (Székely and Rizzo 2013) and extension from marginal to partial DC (Székely and Rizzo 2014). Despite its robustness against model specification, the sample DC statistics used in Li et al. (2012) lacks tail robustness. In this paper, we propose a robust DC via truncation-type robust estimation as a measure for sure screening of ultrahigh-dimensional heavy-tailed data. The proposed new sure screening method based on robust DC (‘RDC’) inherits the same good properties of the original DC-based screening when the data are light-tailed. In addition, it offers the added advantage of robustly estimating DC in the presence of heavy-tailed data, thereby enhancing the screening performance. The proposed measure enjoys the sure screening property with weaker assumptions on the distribution than the original DC-based screening, as justified by our theoretical analysis. Our theorem also reveals a new phenomenon on the optimal choice of robustification parameters. To the best of our knowledge, the proposed method is the only screening method that considers both robustness to model specification and tail robustness. We conducted extensive simulations under different scenarios of heavy-tailedness to demonstrate the advantage of our proposed procedure as compared to existing model-based or model-free screening procedures. The proposed screening procedure was also applied to the high-dimensional heavy-tailed RNA-seq data of The Cancer Genome Atlas (TCGA) pancreatic cancer cohort in prioritizing the most essential and biologically meaningful genes predictive of MAPK1 protein expression critical to pancreatic cancer. To boost the performance of RDC, we further develop a new FDR control procedure. Numerous studies have addressed FDR control in screening procedures (e.g., Zhu et al. (2011); Barut et al. 2016; Zhao and Li 2012); see Tong et al. (2023); Guo et al. (2023) for more comprehensive reviews. However, many of these approaches either lack theoretical guarantees or are specifically tailored to particular screening methods. Recently, Liu et al. (2022) proposed a framework for feature screening with FDR control based on knockoff features. In parallel, Guo et al. (2023) introduced a general framework for FDR-controlled selection via data splitting, referred to as REflection via Data Splitting (REDS). Building on this, Tong et al. (2023) achieved FDR control by applying the REDS method in conjunction with their proposed measure of conditional independence. We adopt the framework of REDS and propose the REDS for controlling FDR of RDC (called ‘RDC-REDS’ in short). Our approach can be implemented in a publicly available R package RobustDC on the GitHub repository: https://github.com/kehongjie/RobustDC.

The rest of the paper is organized as follows. In Section 2, we first introduce the distance correlation measure and the adaptive Huber-type method and then propose our robust distance correlation measure for feature screening in ultrahigh-dimensional data. We also provide the nonasymptotic theoretical results of the proposed measure and show the sure screening property. In Section 3, we propose the REDS for controlling FDR of RDC and show that the related procedure can control the FDR with theoretical guarantees. We conduct extensive simulations in Section 4 to show the strength of our method. In Section 5, we apply our method to an RNA-seq study in pancreatic cancer. The proofs of the main theorems, along with a discussion of potential extensions of the method, are relegated to the Supporting Information.

## 2 | Methods

### 2.1 | Independence Screening Using Distance Correlation

Székely et al. (2007) first introduced the DC measure to generalize the idea of correlation to characterize the joint dependence of any two random vectors. Denote by $\phi_{x}(t)$ and $\phi_{y}(s)$ the respective characteristic functions of two random vectors x and y and $\phi_{x,y}(t,s)$ be the joint characteristic function. The nonnegative distance covariance between x and y with finite first moments is defined as follows:

$${\text{dcov}}^{2}\left(x,y\right)=\int_{R^{d_{x}+d_{y}}}\frac{{\left\|\phi_{x,y}\left(t,s\right)-\phi_{x}\left(t\right)\phi_{y}\left(s\right)\right\|}^{2}}{c_{d_{x}}c_{d_{y}}\|t{\|}_{d_{x}}^{1+d_{x}}\|s{\|}_{d_{y}}^{1+d_{y}}}\text{dtds},$$

where $d_{x}$ and $d_{y}$ are the dimensions of x and $y,c_{d}=\pi^{(1+d)/2}/\Gamma ((1+d)/2)$ with Γ(⋅) being the Gamma function and $\|a{\|}_{d}$ and ‖ϕ‖ represent the Euclidean norm of $a\in R^{d}$ and norm of the complex-valued function ϕ, respectively. Like the Pearson correlation, the nonnegative DC between x and y with finite first moments is defined as $\text{dc}(x,y)=\text{dcov}(x,y)/\sqrt{\text{dcov}(x,x)\text{dcov}(y,y)}$.

Further, Székely et al. (2007) provided an equivalent representation of ${\text{dcov}}^{2}(x,y):{\text{dcov}}^{2}(x,y)=S_{1}+S_{2}-2S_{3}$, where $S_{1},S_{2}$ and $S_{3}$ are defined as follows: $S_{1}=E\left\{{\left\|x-x^{\prime }\right\|}_{d_{x}}{\left\|y-y^{\prime }\right\|}_{d_{y}}\right\},S_{2}=E\left\{||x-x^{\prime }|{|}_{d_{x}}\right\}E\left\{||y-y^{\prime }|{|}_{d_{y}}\right\}$ and $S_{3}=E\left\{E\left(\|x-x^{\prime }{\|}_{d_{x}}|x\right)\right.\left.E\left({\left\|y-y^{\prime }\right\|}_{d_{y}}|y\right)\right\},\left(x^{\prime },y^{\prime }\right)$ is an independent copy of (x,y).

This has motivated the calculation of sample distance covariance by replacing $S_{1},S_{2}$ and $S_{3}$ with the corresponding empirical estimation:

$${\hat{\text{dcov}}}^{2}(x,y)={\hat{S}}_{1}+{\hat{S}}_{2}-2{\hat{S}}_{3},$$

where ${\hat{S}}_{1}=\frac{1}{n^{2}}\sum_{k=1}^{n}\sum_{l=1}^{n}{\left\|x_{k}-x_{l}\right\|}_{d_{x}}{\left\|y_{k}-y_{l}\right\|}_{d_{y}},{\hat{S}}_{2}=\frac{1}{n^{2}}\sum_{k=1}^{n}\sum_{l=1}^{n}{\left\|x_{k}-x_{l}\right\|}_{d_{x}}\frac{1}{n^{2}}\sum_{k=1}^{n}\sum_{l=1}^{n}{\left\|y_{k}-y_{l}\right\|}_{d_{y}}$ and ${\hat{S}}_{3}=\frac{1}{n^{3}}\sum_{k=1}^{n}\sum_{l=1}^{n}\sum_{m=1}^{n}{\left\|x_{k}-x_{l}\right\|}_{d_{x}}{\left\|y_{m}-y_{l}\right\|}_{d_{y}}$. When $d_{x}=d_{y}=1$, they are simplified to ${\hat{S}}_{1}=\frac{1}{n^{2}}\sum_{k=1}^{n}\sum_{l=1}^{n}\left|X_{k}-X_{l}\right|\left|Y_{k}-Y_{l}\right|,{\hat{S}}_{2}=\frac{1}{n^{2}}\sum_{k=1}^{n}\sum_{l=1}^{n}\left|X_{k}-X_{l}\right|\frac{1}{n^{2}}\sum_{k=1}^{n}\sum_{l=1}^{n}\left|Y_{k}-Y_{l}\right|$ and ${\hat{S}}_{3}=\frac{1}{n^{3}}\sum_{k=1}^{n}\sum_{l=1}^{n}\sum_{m=1}^{n}\left|X_{k}-X_{l}\right|\left|Y_{m}-Y_{l}\right|$. Accordingly, the sample DC is defined as follows:

(1) $$\hat{\text{dc}}(x,y)=\frac{\hat{\text{dcov}}(x,y)}{\sqrt{\hat{\text{dcov}}(x,x)\hat{\text{dcov}}(y,y)}}.$$

DC has a few important properties that make it a preferred measure for variable screening: (1) DC is monotonically increasing in the Pearson correlation under normality assumption; thus, using DC for screening is equivalent to using the Pearson correlation for linear regression; (2) DC is equal to zero if and only if x and y are independent; thus, DC is more effective than the Pearson correlation when there exists nonlinear relationship between two random variables; (3) DC measures the dependency between two random vectors so it allows for both multivariate responses $\left(d_{y}>1\right)$ and group-wise predictors $\left(d_{x}>1\right)$, either continuous or discrete; (4) lastly, screening based on DC is model-free without specifying a regression model between the response and the predictors.

Without loss of generality, consider the case with the univariate response, denote by Y the univariate response and $x={\left(X_{1},\ldots ,X_{p}\right)}^{⊤}$ the predictor vector, where p=p(n) is the number of features. For ultrahigh-dimensional data, the dimensionality p grows at an exponential rate of the sample size n. Let $\omega_{j}={\text{dc}}^{2}\left(X_{j},Y\right)$ denote the squared distance correlation. Li et al. (2012) proposed to use its estimator, ${\hat{\omega }}_{j}={\hat{\text{dc}}}^{2}\left(X_{j},Y\right)$, as a measure in independence screening of ultrahigh-dimensional data. They selected important features with large ${\hat{\omega }}_{j}$ as follows: $\left\{j:{\hat{\omega }}_{j}\geq cn^{-\kappa },1\leq j\leq p\right\}$, where c and κ are prespecified threshold values theoretically defined. They also showed the sure screening property of their procedure with the assumption that both x and Y have subexponential tails.

### 2.2 | Huber Loss and Robust Distance Correlation for SIS

Heavy-tailedness is one prominent feature of high-dimensional data that has brought new challenges to conventional statistical methods. In the presence of heavy-tailed distributions, there is a nonnegligible probability that observations are sampled far away from the population mean, causing the empirical mean to seriously deviate from the true mean (Catoni 2012). To tackle heavy-tailed errors, many methods in the literature conducted least absolute deviation (LAD) regression, but they essentially alter the problem by estimating the median or quantiles instead of the mean. In most cases, however, the mean, rather than the median, is the quantity of interest, for example, in the estimation of DC.

Alternatively, one can consider the Huber loss as another way of robustification to adapt for different magnitudes of errors. The Huber loss $l_{\tau }(.)$ (Huber 1964) is defined as follows:

$$l_{\tau }(x)=\left\{\begin{array}{ll} x^{2}/2, & \text{if}\;|x|\leq \tau ,\\ \tau |x|-\tau^{2}/2, & \text{if}\;|x|>\tau , \end{array}\right.$$

where τ>0 is called the robustification parameter. Two extremes of the Huber loss are the least squares and the LAD as τ=∞ and τ=0 respectively. When a majority of |x|’s are small compared to τ, it simplifies to the common squared error loss. In the presence of heavy-tailed errors, the Huber loss downweights large |x|, that is, potential outliers, to introduce robustness. In general, the minimizer becomes more robust but also more biased from the model parameters as τ decreases, so the robustification parameter τ controls the trade-off between bias and robustness. One major task in the development of Huber-type robust methods is to choose an appropriate τ so that we can achieve an optimal trade-off.

Fan et al. (2017) considered high-dimensional regression with coefficients in an $\ell_{q}$ ball (0<q≤1) and proposed a modified Huber’s procedure to obtain a robust estimator of coefficients with the robustification parameter adapted to the sample size and dimension. Sun et al. (2020) further proposed the adaptive Huber regression for robust estimation and inference, allowing q=0 and a more general moment condition of the error in both low and high dimensions. In a similar vein, Ke et al. (2019) directly considered a simple truncated estimator $\psi_{\tau }(x)=(|x|\wedge \tau )\text{sgn}(x)$ for covariance estimation and also proposed simple automatic data-driven tuning scheme to find the optimal robustification parameter for the computation of the robust estimators.

In spite of the nice properties and being model-free, the sample DC in Equation (1) used by Székely et al. (2007) and Li et al. (2012) is not robust against the heavy-tailedness of the data. When the underlying distribution is heavy-tailed, the observations might be sampled far away from the population mean. As a result, the individual sample DC will not be concentrated around the truth which directly impacts its screening performance, given that there are many DC estimates to be considered. To guarantee the tail-robustness in the estimation of DC, we propose a robust estimate of DC (called ‘Robust DC’ or ‘RDC’ in short) by developing some truncated estimators ${\overset{ˆ}{S}}_{1}^{\tau_{x},\tau_{y}},{\hat{S}}_{2}^{\tau_{x}^{\prime },\tau_{y}^{\prime }}$ and ${\overset{ˆ}{S}}_{3}^{\tau_{x},\tau_{y}}$. Suppose we have n i.i.d. samples of two random variables of interest, Y and X, the robust distance covariance and correlation estimates are defined as follows:

(2) $${\hat{\text{rdcov}}}^{2}\left(Y,X\right)={\hat{S}}_{1}^{\tau_{x},\tau_{y}}+{\hat{S}}_{2}^{\tau_{x}^{\prime },\tau_{y}^{\prime }}-2{\hat{S}}_{3}^{\tau_{x},\tau_{y}};\;\hat{\text{rdc}}\left(Y,X\right)=\frac{\hat{\text{rdcov}}\left(Y,X\right)}{\sqrt{\hat{\text{rdcov}}\left(Y,Y\right)\hat{\text{rdcov}}\left(X,X\right)}},$$

where we have that ${\hat{S}}_{1}^{\tau_{x},\tau_{y}}=\frac{1}{n^{2}}\sum_{k=1}^{n}\sum_{l=1}^{n}|\psi_{\tau_{y}}\left(Y_{k}-Y_{l}\right)|\left|\psi_{\tau_{x}}(X_{k}-X_{l})\right|,{\hat{S}}_{2}^{\tau_{x}^{\prime },\tau_{y}^{\prime }}=\frac{1}{n^{2}}\sum_{k=1}^{n}\sum_{l=1}^{n}|\psi_{\tau_{x}^{\prime }}(X_{k}-X_{l})|\times \frac{1}{n^{2}}\sum_{k=1}^{n}\sum_{l=1}^{n}|\psi_{\tau_{y}^{\prime }}\left(Y_{k}-\right.\left.Y_{l}\right)|$ and ${\hat{S}}_{3}^{\tau_{x},\tau_{y}}=\frac{1}{n^{3}}\sum_{k=1}^{n}\sum_{l=1}^{n}\sum_{m=1}^{n}|\psi_{\tau_{y}}\left(Y_{k}-Y_{l}\right)||\psi_{\tau_{x}}\left(X_{m}-X_{l}\right)|$. We have the truncation operator $\psi_{\tau }(u)=(|u|\wedge \tau )\text{sgn}(u)$, with τ being the robustification parameter. Although the idea of truncation is not new in the literature, the selection of different robustification parameters demonstrates novel phenomena. As justified in our theoretical results, we can show that under finite second moment condition, $\left|{\hat{S}}_{2}^{\tau \prime_{x},\tau \prime_{y}}-S_{2}\right|$ is of the order $\left(\frac{\tau_{x}^{\prime }\vee \tau_{y}^{\prime }}{n}+\frac{1}{\tau_{x}^{\prime }}\vee \frac{1}{\tau_{y}^{\prime }}\right)$, where $\frac{1}{\tau_{x}^{\prime }}\vee \frac{1}{\tau_{y}^{\prime }}$ is referred to as the robustification bias. Consequently, both $\tau_{y}^{\prime }$ and $\tau_{x}^{\prime }$ are chosen to scale at the rate of $n^{1/2}$ to reach an optimal trade-off between robustness and bias. Note that the scale of robustification parameter in this case is consistent with established literature (see, e.g., Sun et al. 2020). However, as for $\tau_{x}$ and $\tau_{y}$, their scaling is different from the conventional $n^{1/2}$. To be more specific, it can be seen from Proposition 1, that under slightly higher moment condition, both $\left|{\overset{ˆ}{S}}_{1}^{\tau_{x},\tau_{y}}-S_{1}\right|$ and $\left|{\overset{ˆ}{S}}_{3}^{\tau_{x},\tau_{y}}-S_{3}\right|$ are of the order $\left(\frac{\tau_{x}\tau_{y}}{n}+\frac{1}{\tau_{x}^{2}}\vee \frac{1}{\tau_{y}^{2}}\right)$. Therefore, an optimal choice of $\tau_{x}$ and $\tau_{y}$ should be at the rate of $n^{1/4}$. More discussions on the rate of the robustification parameters can be found in Section 2.4. One may apply the truncation operator once directly to each summand of ${\hat{S}}_{1},{\hat{S}}_{3}$, but this method leads to tuning more parameters. To elaborate, with p predictors and one response variable, the current approach involves only p+1 robustification parameters in the computation of robustified ${\hat{S}}_{1},{\hat{S}}_{3}$ for the distance covariance estimates between the response and each predictor. To be more specific, we need to tune one robustification parameter for the response and one parameter for each predictor, respectively. In contrast, the aforementioned approach requires the tuning of 2p parameters in the equivalent calculation. In order to get the robust estimates of ${\hat{S}}_{1},{\hat{S}}_{3}$ between one predictor and response, we have to tune two parameters. Since there are p predictors, this results in 2p tuning parameters in total. In addition, it is worth noting that one can apply the Huber loss to obtain robust M-estimators for $S_{1},S_{2}$ and $S_{3}$ separately, resulting in a slightly smaller bias, as discussed in Section 3.3 of Ke et al. (2019). This corresponds to directly truncating each summand in ${\hat{S}}_{1}$ and ${\hat{S}}_{3}$. However, as discussed above, this approach is less computationally efficient than the truncation strategy used in RDC. Therefore, we recommend our RDC-based robust estimator over the Huber-loss-based alternative due to its simplicity and computational efficiency. Details on the theoretical properties of optimal choice of τ and how to determine it in a data-driven fashion will be discussed in Sections 2.3 and 2.4.

Based on the predictor vector $x={\left(X_{1},\ldots ,X_{p}\right)}^{⊤}$ and the response variable Y, we will use ${\hat{\omega }}_{j}^{\tau }={\hat{\text{rdc}}}^{2}\left(X_{j},Y\right)$ as a marginal utility to rank the relative importance of $X_{j}$ and perform independence screening. The features with the largest ${\hat{\omega }}_{j}^{\tau }$ are kept after screening, that is, the set $\left\{j:{\hat{\omega }}_{j}^{\tau }\geq K\sqrt{\text{log}p/n},1\leq j\leq p\right\}$, where K is a sufficiently large constant. In practice, as for all screening methods, the top number of features to be left, that is, d, is a key tuning parameter that empirically balances the trade-off between false negative and false positive errors. In this paper, we followed Fan and Lv (2008) and chose d=⌊n/logn⌋.

*Remark* 1. Multivariate responses or grouped predictors are common in modern statistical applications. For example, many complex diseases consist of a large number of highly correlated clinical phenotypes that are associated with pleiotropic genes or genetic variants. Li et al. (2012) showed in simulations that the DC can be successfully used to screen for multivariate responses or grouped predictors. Though we introduced the method using univariate response and predictor notation, our method is built based on the original DC and thus is readily extended to perform robust screening for multivariate responses with $d_{y}>1$ or grouped predictors with $d_{x}>1$. The tuning of the robustification parameter will remain the same in the multivariate case.

### 2.3 | Tuning of the Robustification Parameter and Algorithm RDC-SIS

A data-driven procedure is proposed to automatically tune the robustification parameters. Define the pairwise difference by a generic notation $\left\{Z_{1},\ldots ,Z_{N}\right\}=\left\{\left|Y_{1}-Y_{2}\right|,\ldots ,\left|Y_{n-1}-Y_{n}\right|\right\}$ for the response Y or $\left\{\left|X_{1j}-X_{2j}\right|,\ldots ,\left|X_{(n-1)j}-X_{nj}\right|\right\}$ for the jth predictor of x, where N=n2. In theory, as specified in Theorem 1, we have that $\tau^{\prime }={\left[E\left(Z_{1}^{2}\right)\right]}^{1/2}(n/t{)}^{1/2}$, where $\tau^{\prime }$ represents either $\tau_{x}^{\prime }$ or $\tau_{y}^{\prime }$, and t is some quantity specified in the theorem. However, $E\left(Z_{1}^{2}\right)$ itself is unknown and needs to be estimated. The empirical mean $\sum_{i=1}^{N}Z_{i}^{2}/N$, as a naive estimator of $E\left(Z_{1}^{2}\right)$, can suffer from overestimation, if $Z_{i}$’s are of heavy tail. Hence, we resort to $\frac{1}{N}\sum_{i=1}^{N}Z_{i}^{2}\wedge \tau^{\prime 2}$ to get a robust estimate of $E\left(Z_{1}^{2}\right)$. Intuitively, a good choice of $\tau^{\prime }$ for $Z_{i}$ should make $\frac{1}{N}\sum_{i=1}^{N}Z_{i}^{2}\wedge \tau^{\prime 2}$ a reasonable estimator. By plugging this estimator into the aforementioned formula of $\tau^{\prime }$, we end up with the following equation:

(3) $$\frac{1}{N}\sum_{i=1}^{N}\frac{\left(Z_{i}^{2}\wedge \tau^{\prime 2}\right)}{\tau^{\prime 2}}=\frac{t}{n}.$$

Subsequently, ‘ideal’ choice of $\tau^{\prime }$ can be solved from Equation (3). Following the similar argument in Ke et al. (2019), it can be shown that the solution is unique, when $t<\frac{n}{N}\sum_{i=1}^{N}1\left\{Z_{i}\neq 0\right\}$. Additional details, including the theoretical properties of this tuning method, can be found in Ke et al. (2019). Analogously, as demonstrated in Theorem 1, we have $\tau ={\left[E\left(Z_{1}^{4}\right)\right]}^{1/4}(n/t{)}^{1/4}$. By replacing $E\left(Z_{1}^{4}\right)$ with its robust estimator $\frac{1}{N}\sum_{i=1}^{N}Z_{i}^{4}\wedge \tau^{4}$, we end up with Equation (4) below:

(4) $$\frac{1}{N}\sum_{i=1}^{N}\frac{\left(Z_{i}^{4}\wedge \tau^{4}\right)}{\tau^{4}}=\frac{t}{n}.$$

Analogously, a good choice of τ can be solved from this equation. Again, it can be shown that the solution is unique if $t<\frac{n}{N}\sum_{i=1}^{N}1\left\{Z_{i}\neq 0\right\}$.

In practice, we let t=Clogp, where for both $\tau^{\prime }$ and τ, the choice of C is the same for all $X_{j}$’s (1≤j≤p) and Y. In our initial experiments, we observed that when the parameter C is set to values within a practical range, the predictors selected remain consistent. In the package, we provide a default choice as well as some alternative data-driven choice. For the application to real data, we set C=0.2. From the above, we can get optimal estimates of τ and $\tau^{\prime }$ for the response and each of the predictor variables, respectively. We then plug τ and $\tau^{\prime }$ estimates back into Equation (2) to compute each of ${\overset{ˆ}{S}}_{1}^{\tau_{x},\tau_{y}},{\overset{ˆ}{S}}_{2}^{\tau_{x}^{\prime },\tau_{y}^{\prime }}$ and ${\overset{ˆ}{S}}_{3}^{\tau_{x},\tau_{y}}$ and finally solve for $\hat{\text{rdcov}}\left(X_{j},Y\right)$ and $\hat{\text{rdc}}\left(X_{j},Y\right)$. Below is an algorithm to estimate RDC and perform sure screening, where we use $\tau_{j}$ and $\tau_{j}^{\prime }$ to denote the associated robustification parameters for $X_{j}$. Following sure screening, one can perform a second-stage variable selection by commonly used regularization methods such as Lasso, Elastic net or SCAD, to select the final set of predictors. Alternatively, we may refer to the original SIS screening method in Fan and Lv (2008) and propose an iterative version of RDC-SIS by removing variables in a stepwise fashion. In Section 3, we further develop a new FDR control procedure based on the proposed RDC.

### 2.4 | Theoretical Properties for Sure Screening

Let F(Y∣x) be the conditional distribution function of Y given x. In analogy to Li et al. (2012), we define the index set of active predictors as $𝒟=\left\{j:F(Y|x)\right.\text{depends}\;\text{on}\left.X_{j}\right\}$. Let ${𝒟}^{c}=\{1,\ldots ,p\}/𝒟$. It is evident to see that conditioning on $x_{𝒟},Y$ and $x_{{𝒟}^{c}}$ are independent. Therefore, $x_{𝒟}$ are the predictors of interest. Subsequently, we define its estimated counterpart as $\hat{𝒟}=\left\{j:{\hat{\omega }}_{j}^{\tau }\geq K\sqrt{\text{log}p/n}\right.,\;\text{for}\;\left.1\leq j\leq p\right\}$, where K is a sufficiently large constant. Then, under some mild regularity conditions below, we show in Theorem 1 that the proposed RDC has the property of SIS.

**Assumption 1.** Throughout, we use $\left(\tilde{Y},{\tilde{X}}_{j}\right)$ to denote an i.i.d. copy of $\left(Y,X_{j}\right)$. There exists a universal constant $\tilde{C}$, such that ${\left[E\left(|Y-\tilde{Y}{|}^{4}\right)\right]}^{1/4}\leq \tilde{C}$ and $\underset{1\leq j\leq p}{\text{max}}{\left[E\left({\left|X_{j}-{\tilde{X}}_{j}\right|}^{4}\right)\right]}^{1/4}\leq \tilde{C}$.

**Assumption 2.** There exists a universal constant $\tilde{c}$, such that $\underset{1\leq j\leq p}{\text{min}}\;{\text{dcov}}^{2}\left(X_{j},X_{j}\right)\geq \tilde{c}$ and ${\text{dcov}}^{2}(Y,Y)\geq \tilde{c}$.

**Assumption 3.** The minimum DC of active predictors satisfies $\underset{j\in 𝒟}{\text{min}}\;\omega_{j}\geq 2K\sqrt{\text{log}p/n}$, where K is a sufficiently large constant and $\sqrt{\text{log}p/n}=o(1)$.

It is worth noting that Assumption 1 posits bounded fourth moments, paralleling the conditions presented in Ke et al. (2019). This is because in both cases, we need to robustify the covariance estimates, thereby necessitating a more stringent requirement for finite fourth moments. In contrast, within the context of robust mean estimation, the assumption of a finite second moment is considered sufficient. The distance variances of Y and $X_{j}$’s are assumed to be bounded away from zero in Assumption 2, which is implicitly assumed in Li et al. (2012). Although this assumption can be relaxed, we still impose it here to simplify the proof. Assumption 3 is parallel to Condition (C2) in Li et al. (2012), where they have that $\underset{j\in 𝒟}{\text{min}}\;\omega_{j}\geq 2cn^{-\kappa }$ with c>0 and 0≤κ<1/2. Consequently, they impose a sample requirement with $\text{log}p=o\left(n^{1-2\kappa }\right)$ in the ultra-high dimensional setting. It should be noted that their sample requirement implies the one demonstrated in our Assumption 3. Therefore, we are working with a slightly weaker assumption.

**ALGORITHM 1 |:** RDC-SIS method

**Input:** Predictor vectors $x_{i}={\left(X_{i1},\ldots ,X_{ip}\right)}^{⊤}\in R^{p}$ and the outcome variable $Y_{i}$ for i=1,…,n; pre-specified C and d.
**Estimation:** Data-adaptive estimation of RDC
1: Calculate the pairwise difference of each predictor variable $\left\{Z_{j1},\ldots ,Z_{jN}\right\},1\leq j\leq p$ and the outcome variable $\left\{Z_{y1},\ldots ,Z_{yN}\right\}$.
2: Given t=Clogp, solve $\left(\tau_{j},\tau_{j}^{\prime }\right)$, and $\left(\tau_{y},\tau_{y}^{\prime }\right)$ for each predictor variable and the outcome variable from Equations (3)–(4).
3: For each predictor 1≤j≤p, compute the truncated versions of ${\hat{S}}_{1}^{\tau_{j},\tau_{y}},{\hat{S}}_{2}^{\tau_{j}^{\prime },\tau_{y}^{\prime }}$ and ${\hat{S}}_{3}^{\tau_{j},\tau_{y}}$ and thus $\hat{\text{rdcov}}\left(Y,X_{j}\right)$ and $\hat{\text{rdc}}\left(Y,X_{j}\right)$ from Equation (2).
**Screening:** Compute ${\hat{\omega }}_{j}^{\tau }={\hat{\text{rdc}}}^{2}\left(Y,X_{j}\right)$ and sort all features by ${\hat{\omega }}_{j}^{\tau }$ from the largest to the smallest.
**Output:** The top d features ranked by ${\hat{\omega }}_{j}^{\tau }$.

As briefly discussed in Section 2.2, $\tau_{x},\tau_{y}$ and $\tau_{x}^{\prime },\tau_{y}^{\prime }$ scale at different order. The following Proposition 1 demonstrates the explicit robustification bias of ${\overset{ˆ}{S}}_{1}^{\tau_{x},\tau_{y}},{\hat{S}}_{2}^{\tau_{x}^{\prime },\tau_{y}^{\prime }}$ and ${\hat{S}}_{3}^{\tau_{x},\tau_{y}}$. This, in turn, indicates the optimal choice of truncation parameters necessary to achieve the variance-bias trade-off.

**Proposition 1.** Robustification bias For any j∈{1,…,p}, suppose $\left(Y,X_{j}\right),\left(\tilde{Y},{\tilde{X}}_{j}\right)$ and $\left(\overset{ˇ}{Y},{\overset{ˇ}{X}}_{j}\right)$ are three independent copies. We define the following quantities: $S_{1}^{j}=E\left(|Y-\tilde{Y}|\left|X_{j}-{\tilde{X}}_{j}\right|\right),S_{1}^{*j}=E\left(\psi_{\tau_{y}}(|Y-\tilde{Y}|)\psi_{\tau_{j}}\left(\left|X_{j}-{\tilde{X}}_{j}\right|\right)\right),S_{2,1}^{j}=E(|Y-\tilde{Y}|),S_{2,1}^{*j}=E(\psi_{\tau_{y}^{\prime }}(|Y-\tilde{Y}|)),S_{2,2}^{j}=E\left(\left|X_{j}-{\tilde{X}}_{j}\right|\right),S_{2,2}^{*j}=E\left(\psi_{\tau_{j}^{\prime }}\left(\left|X_{j}-{\tilde{X}}_{j}\right|\right)\right),S_{3}^{j}=E(|\tilde{Y}-Y||{\overset{ˇ}{X}}_{j}-X_{j}|)$ and $S_{3}^{*j}=E\left(\psi_{\tau_{y}}(|\tilde{Y}-Y|)\psi_{\tau_{j}}\left(\left|{\overset{ˇ}{X}}_{j}-X_{j}\right|\right)\right)$. Then, we have that under Assumption 1,

$$S_{1}^{j}-S_{1}^{*j}\leq \frac{4}{27}\left(\frac{1}{\tau_{y}^{2}}{\left[E\left(|Y-\tilde{Y}{|}^{4}\right)\right]}^{3/4}{\left[E\left({\left|X-{\tilde{X}}_{j}\right|}^{4}\right)\right]}^{1/4}\right.\left.+\frac{1}{\tau_{j}^{2}}{\left[E\left(|Y-\tilde{Y}{|}^{4}\right)\right]}^{1/4}{\left[E\left({\left|X-{\tilde{X}}_{j}\right|}^{4}\right)\right]}^{3/4}\right),\;S_{2,1}^{j}-S_{2,1}^{*j}\leq \frac{1}{4\tau_{y}^{\prime }}E\left(|Y-\tilde{Y}{|}^{2}\right),S_{2,2}^{j}-S_{2,2}^{*j}\leq \frac{1}{4\tau_{j}^{\prime }}E\left({\left|X_{j}-{\tilde{X}}_{j}\right|}^{2}\right),\;S_{3}^{j}-S_{3}^{*j}\leq \frac{4}{27}\left(\frac{1}{\tau_{y}^{2}}{\left[E\left(|Y-\tilde{Y}{|}^{4}\right)\right]}^{3/4}{\left[E\left({\left|X-{\overset{ˇ}{X}}_{j}\right|}^{4}\right)\right]}^{1/4}\right.\left.+\frac{1}{\tau_{j}^{2}}{\left[E\left(|Y-\tilde{Y}{|}^{4}\right)\right]}^{1/4}{\left[E\left({\left|X-{\overset{ˇ}{X}}_{j}\right|}^{4}\right)\right]}^{3/4}\right).$$

Proposition 1 implies that the robustification bias for both ${\hat{S}}_{1}^{\tau_{j},\tau_{y}}$ and ${\hat{S}}_{3}^{\tau_{j},\tau_{y}}$ are of the order $\frac{1}{\tau_{j}^{2}}\vee \frac{1}{\tau_{y}^{2}}$. Due to the boundedness, their standard deviations are of the order $\frac{\tau_{j}\tau_{y}}{n}$. Consequently, we can conclude $\left|{\hat{S}}_{1}^{\tau_{j},\tau_{y}}-S_{1}^{j}\right|$ and $\left|{\hat{S}}_{3}^{\tau_{j},\tau_{y}}-S_{3}^{j}\right|$ scale at the rate of $\left(\frac{\tau_{j}\tau_{y}}{n}+\frac{1}{\tau_{j}^{2}}\vee \frac{1}{\tau_{y}^{2}}\right)$. Therefore, it is optimal to make $\tau_{j}$ and $\tau_{y}$ scaled at the order of $n^{1/4}$. Similarly, one can find that $\tau_{j}^{\prime }$ and $\tau_{y}^{\prime }$ should be of the order $n^{1/2}$. With the properly chosen truncation parameters, the following main theorem establishes the sure screening property for RDC-SIS.

**Theorem 1.** Suppose the truncation levels are set to be $\tau_{y}={\left[E\left(|Y-\tilde{Y}{|}^{4}\right)\right]}^{1/4}(n/t{)}^{1/4},\tau_{y}^{\prime }={\left[E\left(|Y-\tilde{Y}{|}^{2}\right)\right]}^{1/2}(n/t{)}^{1/2},\tau_{j}=[E{\left.\left({\left|X_{j}-{\tilde{X}}_{j}\right|}^{4}\right)\right]}^{1/4}(n/t{)}^{1/4}$, and $\tau_{j}^{\prime }={\left[E\left({\left|X_{j}-{\tilde{X}}_{j}\right|}^{2}\right)\right]}^{1/2}(n/t{)}^{1/2}$, where t=o(n) is some quantity to be chosen. Then under Assumptions 1 and 2, we have that

$$P\left(\underset{1\leq j\leq p}{\text{max}}\left|{\hat{\omega }}_{j}^{\tau }-\omega_{j}\right|\geq C_{v}\sqrt{\frac{t}{n}}\right)\leq C_{v}^{\prime }p\text{exp}\{-t\},$$

where $C_{v}$ and $C_{v}^{\prime }$ are two universal constants, which can be traced from the proof. Subsequently, by picking t=Clogp with C>1, under Assumption 3, we obtain that for large $n,P\left(\underset{1\leq j\leq p}{\text{max}}\left|{\hat{\omega }}_{j}^{\tau }-\omega_{j}\right|\geq K\sqrt{\text{log}\;p/n}\right)\leq C_{v}^{\prime }p^{1-C}$. Furthermore, we also conclude that $P(𝒟\subseteq \hat{𝒟})\geq 1-C_{v}^{\prime }sp^{-C}$, where s is the cardinality of 𝒟.

## 3 | False Discovery Rate Control With REDS

We adopt the framework of Guo et al. (2023) and construct the REDS statistic in the context of our RDC estimator as follows. Data are randomly split into two groups ${𝒲}_{1}$ and ${𝒲}_{2}$ of unequal size, with $n_{1}=n(T-1)/T$ and $n_{2}=n/T$ (in practice, we set T=3). For each 1≤j≤p, we compute the RDC estimators ${\overset{ˆ}{\omega }}_{j1}^{\tau }$ and ${\hat{\omega }}_{j2}^{\tau }$ based on ${𝒲}_{1}$ and ${𝒲}_{2}$, respectively. The resulting REDS statistic (‘RDC-REDS’) used for FDR control is defined as follows:

(5) $$W_{j}=\text{sgn}\left(\sqrt{n_{1}}{\hat{\omega }}_{j1}^{\tau }-\sqrt{n_{2}}{\hat{\omega }}_{j2}^{\tau }\right)\left(\sqrt{n_{1}}{\hat{\omega }}_{j1}^{\tau }\vee \sqrt{n_{2}}{\hat{\omega }}_{j2}^{\tau }\right).$$

Here, we use $W_{j}$ as a new marginal utility to rank the relative importance of $X_{j}$. Given the REDS statistics $W_{j}$’s, the false discovery proportion (FDP) at threshold level t is defined as $\text{FDP}(t)=\#\left\{j\in {𝒟}^{c}:W_{j}\geq t\right\}/\left(\#\left\{j:W_{j}\geq t\right\}\vee 1\right)$. Accordingly, the FDR(t) is defined as the expectation of FDP(t), that is, FDR(t)=E[FDP(t)]. Our goal is to identify a threshold t such that FDR(t)≤α, for a prespecified level α.

The motivation for constructing the REDS statistics $W_{j}$’s is as follows. For $j\in {𝒟}^{c}$, both ${\sqrt{n}}_{1}{\hat{\omega }}_{j1}^{\tau }$ and ${\sqrt{n}}_{2}{\hat{\omega }}_{j2}^{\tau }$ converge to the same limiting distribution with mean 0. Thus, $W_{j}$ is asymptotically symmetric (see Proposition 2). In contrast, for j∈𝒟, since $n_{1}>n_{2}$, heuristically, we expect with high probability that $\sqrt{n}{\hat{\omega }}_{j1}^{\tau }>{\sqrt{n}}_{2}{\hat{\omega }}_{j2}^{\tau }$, which implies $W_{j}>0$. Based on this observation, the quantity $\#\left\{j:W_{j}\leq -t\right\}$ can be used to approximate $\#\left\{j\in {𝒟}^{c}:W_{j}\geq t\right\}$. Consequently, the data-driven threshold L is chosen as follows:

(6) $$L=\text{inf}\left\{t>0:\frac{1+\#\left\{j:W_{j}\leq -t\right\}}{\#\left\{j:W_{j}\geq t\right\}\vee 1}\leq \alpha \right\}.$$

For the threshold L determined in (6), let $\hat{ℳ}(L)=\left\{j:W_{j}\geq L\right\}$ denote the set of selected predictors as an estimator of 𝒟. The above FDR control procedure based on RDC-REDS is summarized in the following algorithm.

To ensure that the RDC-based REDS statistics in Algorithm 2 yield a set of selected predictors with FDR asymptotically controlled at the target level α, that is, ${\text{limsup}}_{(n,p)\to \infty }\text{FDR}(L)\leq \alpha$, we impose several additional assumptions. Due to space limitations, these assumptions are provided in the Supporting Information. In summary, Assumption S1 imposes stronger moment conditions and a more stringent sample size requirement compared to Assumptions 1 and 3. This is needed to establish the asymptotic normality of $\sqrt{n}{\hat{\omega }}_{j}^{\tau }$ with mean 0 for $j\in {𝒟}^{c}$. In particular, Assumption S1 ensures that the bias introduced by truncation is negligible. Unlike Assumption 3, which requires sufficiently large $\omega_{j}$ values for j∈𝒟 to guarantee sure screening, we instead require $\omega_{j}$ to be relatively small for $j\in {𝒟}^{c}$ so that inactive predictors can be effectively separated from the active ones. Assumption S2 provides a specific condition on the rate at which these $\omega_{j}$’s converge to zero in order to be considered negligible. Assumption S3 addresses the kernel conditions and is used to derive the Berry-Esseen bound for $\sqrt{n}{\hat{\omega }}_{j}^{\tau }$. Finally, Assumption S4 states that the correlations among $W_{j}$’s for $j\in {𝒟}^{c}$ cannot be too strong, as is commonly assumed in the literature.

**ALGORITHM 2 |:** RDC-REDS method

**Input:** Predictor vectors $x_{i}={\left(X_{i1},\ldots ,X_{ip}\right)}^{⊤}\in R^{p}$ and the outcome variable $Y_{i}$ for i=1,…,n.
**Estimation:**
1: Randomly split the data into two groups ${𝒲}_{1}$ and ${𝒲}_{2}$ of unequal size, $n_{1}=n(T-1)/T$ and $n_{2}=n/T$ (by default T=3).
2: For each 1≤j≤p, follow Algorithm 1 to compute ${\overset{ˆ}{\omega }}_{j1}^{\tau }$ and ${\overset{ˆ}{\omega }}_{j2}^{\tau }$ based on ${𝒲}_{1}$ and ${𝒲}_{2}$ respectively, and then construct the RDC-REDS statistics by (5).
3: Choose the threshold L by (6).
**Output:** The selected predictors are $\hat{ℳ}(L)=\left\{j:W_{j}\geq L\right\}$.

Under the additional assumptions outlined above, the $W_{j}$’s for $j\in {𝒟}^{c}$ are asymptotically symmetric. This result is formally stated in the following proposition.

**Proposition 2.** Asymptotic symmetry Under Assumptions S1–S4 from the Supporting Information, along with Assumption 2, for the constructed REDS statistics in Algorithm 2, we have that for any $t>0,{\text{max}}_{j\in {𝒟}^{c}}\left|P\left(W_{j}>t\right)-P\left(W_{j}<-t\right)\right|\to 0$, as (n,p)→∞.

Given the asymptotic symmetry of $W_{j},j\in {𝒟}^{c}$ and the weak dependence assumption among them (as stated in Assumption S4), following similar argument as in Dai et al. (2023), one can show that Algorithm 2 (RDC-REDS) asymptotically controls the FDR at the desired level α. This result is formally presented in Theorem 2.

**Theorem 2.** Assuming the same conditions as in Proposition 2, with the constructed REDS statistics, $W_{j}$’s, in Algorithm 2 and the threshold L from (6) for a target level α, we can conclude $\text{FDP}(L)\leq \alpha +o_{p}(1)$, and ${\text{limsup}}_{(n,p)\to \infty }\text{FDR}(L)\leq \alpha$.

## 4 | Simulation

In this section, we conduct comprehensive Monte Carlo simulations to demonstrate the efficacy of our proposed screening method compared to other existing methods under various scenarios. In addition to RDC method, we include seven other popular sure screening methods for comparison: marginal correlation screening (SIS; Fan and Lv 2008), the original distance correlation screening (DC; Li et al. 2012), sure independent ranking and screening (SIRS; Zhu et al. 2011), fused Kolmogorov filter (FK; Mai and Zou 2015), nonparametric independence screening (NIS; Fan et al. 2011), the Ball correlation based sure screening (BCOR-SIS; Pan et al. 2019) and the stable correlation (SC-SIS, Guo et al. 2022). For the FK method, we consider $G_{i}=2,\ldots ,6$, where $G_{i}$ denotes the number of intervals used to slice the response variable. As for the SC-SIS, the parameter a is set to be 0.5, as suggested by the authors. For each simulation scenario, we replicate for B=100 times and evaluate the screening performances of different methods based on the four criteria below: (i) minimum model size (MMS): the minimum model size to include all active predictors. (ii) ${𝒫}_{1}$: the proportion of replications that include at least one true active predictor for a given model size d. (iii) ${𝒫}_{a}$: the proportion of replications that include all true active predictors for a given model size d. (iv) ${TP}_{d}$: average number of true active predictors identified for a given model size d.

For ${𝒫}_{1}$ and ${𝒫}_{a}$, we set d=⌊n/logn⌋. When calculating $TP_{d}$, we introduce two larger model sizes, $d_{1}=\lfloor 2n/\text{log}n\rfloor$ and $d_{2}=\lfloor 3n/\text{log}n\rfloor$, for a comprehensive evaluation.

### (I).

In the first scenario, we consider the same models as those used in the DC paper (Li et al. 2012). Let $x={\left(X_{1},X_{2},\ldots ,X_{p}\right)}^{⊤}$ denote the vector of predictors. Models (Ia) and (Ib) assume light-tailed distributions, where predictors are normally distributed; Models (Ic) and (Id) assume heavy-tailed distributions, where the predictors follow a t-distribution. In Model (Ie), we further consider the nonlinear relationship between the response and the predictors.

(Ia). $x\sim N\left(0,\Sigma_{p\times p}\right),Y=\alpha_{1}\beta_{1}X_{1}+\alpha_{2}\beta_{2}X_{2}+\alpha_{3}\beta_{3}I\left(X_{12}<0\right)+\alpha_{4}\beta_{4}X_{22}+\epsilon ;$
(Ib). $x\sim N\left(0,\Sigma_{p\times p}\right),Y=\alpha_{1}\beta_{1}X_{1}X_{2}+\alpha_{3}\beta_{2}I\left(X_{12}<0\right)+\alpha_{4}\beta_{3}X_{22}+\epsilon ;$
(Ic). $x\sim t_{3}\left(0,\Sigma_{p\times p}\right),Y=\alpha_{1}\beta_{1}X_{1}+\alpha_{2}\beta_{2}X_{2}+\alpha_{3}\beta_{3}I\left(X_{12}<0\right)+\alpha_{4}\beta_{4}X_{22}+\epsilon ;$
(Id). $x\sim t_{3}\left(0,\Sigma_{p\times p}\right),Y=\alpha_{1}\beta_{1}X_{1}X_{2}+\alpha_{3}\beta_{2}I\left(X_{12}<0\right)+\alpha_{4}\beta_{3}X_{22}+\epsilon ;$
(Ie). $x\sim t_{3}\left(0,\Sigma_{p\times p}\right),Y=\alpha_{1}\beta_{1}X_{1}+\alpha_{2}\beta_{2}X_{2}+\alpha_{3}\beta_{3}I\left(X_{12}<0\right)+\text{exp}\left(\alpha_{4}X_{22}\right)+\epsilon ,$

where $\Sigma_{jj}=1$ and $\Sigma_{jj^{\prime }}=0.5^{\left|j-j^{\prime }\right|}$ for $1\leq j\neq j^{\prime }\leq p$. For j=1,2,3 and 4, we set $\beta_{j}=(-1{)}^{U}(a+|Z|)$, where $a=4\text{log}n/\sqrt{n},U\sim \text{Bern}(0.4)$ and Z~N(0,1). We fix $\left(\alpha_{1},\alpha_{2},\alpha_{3},\alpha_{4}\right)=(2,0.5,3,2)$ and assume the error term ϵ follows the standard normal distribution N(0,1). For all models in scenario (I), we consider n=200 and p=2000.

### (II).

In the second scenario, we further explore alternative heavy-tailed distributions for the predictors and additionally consider heavy-tailed distribution for the response.

(IIa). $x\sim t_{3}\left(0,\Sigma_{p\times p}\right),Y\sim t_{2}\left(x^{⊤}\beta \right)$;
(IIb). $X_{j}$ independently drawn from Pareto(1,1) for $1\leq j\leq p,Y\sim t_{2}\left(x^{⊤}\beta \right)$,

where Σ in (IIa) is set up the same way as in (I). The coefficient vector β is sparse, with only four predictors having nonzero coefficients (2, −2,2, −2). These active predictors (i.e., s=4) are evenly distributed among the p variables. For models in scenario (II), we consider n=100 and p=2000.

### (III).

In the third scenario, we consider multivariate response settings. The predictor x is generated from the same heavy-tailed distributions as in scenario (II), and the response vector $y={\left(Y_{1},Y_{2},Y_{3}\right)}^{⊤}$ is drawn from a multivariate normal distribution with common mean $x^{⊤}\beta$ and covariance matrix $\Sigma_{Y}={\left(\sigma_{Y,kk^{\prime }}\right)}_{3\times 3}$, where $\Sigma_{Y,kk}=1$ and $\Sigma_{Y,kk^{\prime }}=0.5$ for $k\neq k^{\prime }$. The coefficients for the first two predictors are set to be nonzero: $\beta_{1}=\beta_{2}=1$ (i.e., the number of true active predictors is s=2). As in scenario (II), we set n=100 and p=2000.

Table 1 presents the results for the first scenario. RDC-SIS and DC-SIS exhibit comparable performance in settings (Ia) and (Ib), where both the predictors and the response are light-tailed. In contrast, RDC-SIS outperforms DC-SIS in settings (Ic) and (Id), where the predictors follow a t-distribution, as indicated by lower MMS values and higher scores in ${𝒫}_{1},{𝒫}_{a}$ and $TP_{d}$. To evaluate more complex relationships between the response and the predictors, we follow Li et al. (2012) and consider setting (Ie) with an exponential relationship. In this case, Pearson correlation-based SIS fails, while RDC-SIS remains among the best-performing methods. For both scenarios (II) and (III), RDC consistently outperforms nearly all other methods, as shown in Tables 2 and 3.

## 5 | Real Data Application

We applied our method to a real genomic dataset from the Pancreatic Adenocarcinoma (PAAD) cohort of The Cancer Genome Atlas (TCGA), a comprehensive consortium project analysing multiplatform molecular profiles spanning 33 cancer types (Weinstein et al. 2013). PAAD is the most common type of pancreatic cancer and has one of the poorest prognosis among all cancers. Previous studies showed that KRAS mutation and RAS-MAPK signaling pathway are essential to the understanding of pancreatic cancer (Ryan et al. 2014; Furukawa 2015; Raphael et al. 2017). Our objective is to investigate the effects of upstream gene expression on the expression of MAPK1 protein, a key component in the MAPK pathway. We obtained the RNA sequencing (RNA-seq) gene expression data and retrieved the MAPK1 protein expression levels from the Reverse Phase Protein Array (RPPA) of the TCGA PAAD cohort through the Broad Institute’s GDAC Firehose (https://gdac.broadinstitute.org/). The RNA-seq data were quantified in Transcripts Per Million (TPM) values, and the RPPA data comprised continuous intensity values. After filtering out genes with an average TPM of less than 10 in the RNA-seq data and consolidating the samples available from both datasets, we retained 16,191 genes across 116 PAAD cancer cases for our analysis.

We applied our robust DC-based screening method RDC-SIS as well as its competitors to rank and identify genes most predictive of MAPK1 protein expression. Given the absence of ground truth in real data, we used pathway analysis results to validate the top-ranked genes identified by each screening method and to evaluate their feature screening performance accordingly. For a fair comparison, we first took the top 500 genes ranked by each method and conducted a pathway enrichment analysis by Fisher’s exact test for each set, using GO, KEGG, Reactome and Biocarta databases downloaded from MSigDB (Subramanian et al. 2005). Pathways common to the top 200 enriched pathways from each method were considered as functional domains most indicative of the underlying biological truth and treated as a ‘gold standard’ for comparison. We compared the overall performance of different methods by plotting the mean and standard errors of −log10 (p-value) from the enrichment tests of these pathways, with varying numbers of top-ranked features for each screening method. The best method is the one, demonstrating the most significant enrichment in these pathways regardless of the number of features used. The predictive performance of the final set of selected genes, postapplication of the second-stage regularization, is assessed by considering a robust version of mean absolute error (MAE) via the leave-one-out cross-validation: $MAE(\hat{\beta })=\frac{1}{n_{\text{test}}}\sum_{i=1}^{n_{\text{test}}}\left|Y_{i}^{\text{test}}-{\left(x_{i}^{\text{test}}\right)}^{⊤}\hat{\beta }\right|$, where $Y_{i}^{\text{test}}$ and $x_{i}^{\text{test}}$ are the response and predictor variables of the observation in the test data.

**FIGURE 1 |:**
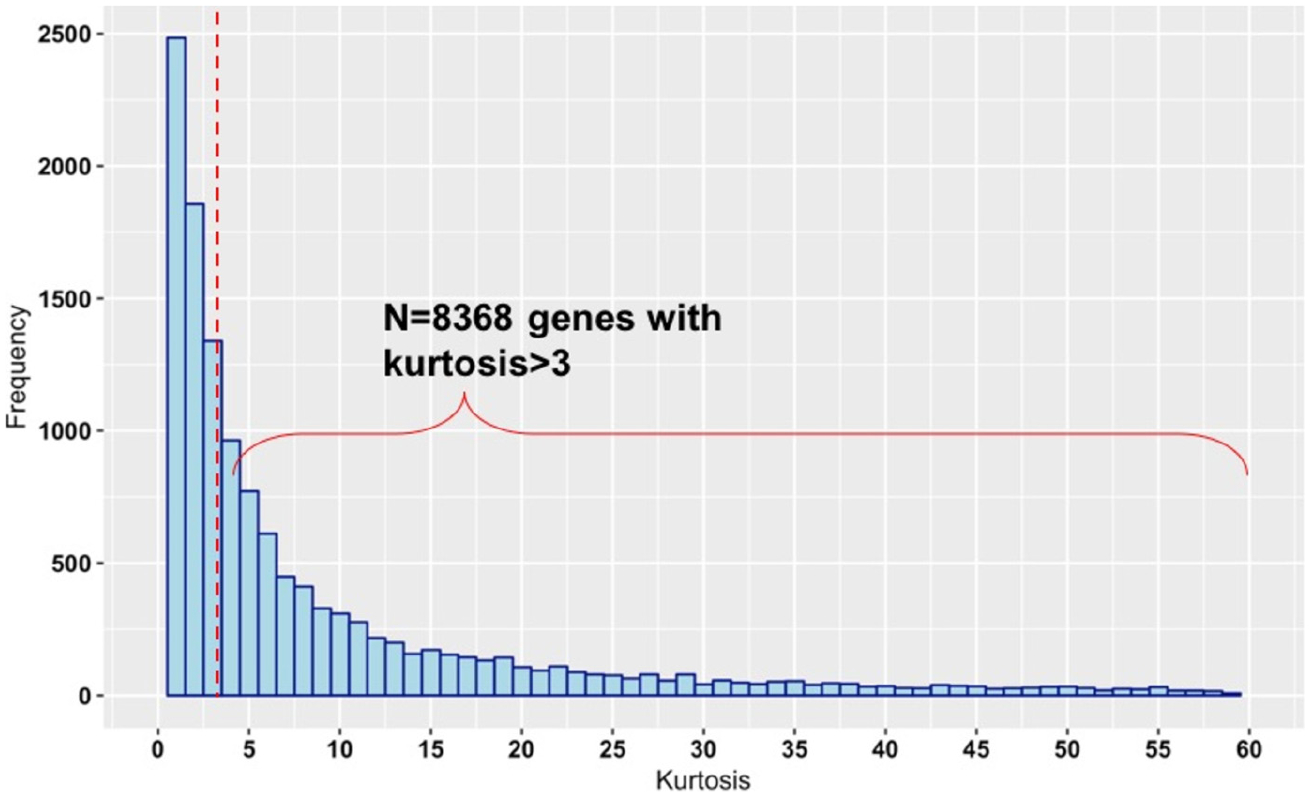
Distribution of kurtosis of expression of all genes. The kurtosis of MAPK1 protein expression is equal to 5.5.

**FIGURE 2 |:**
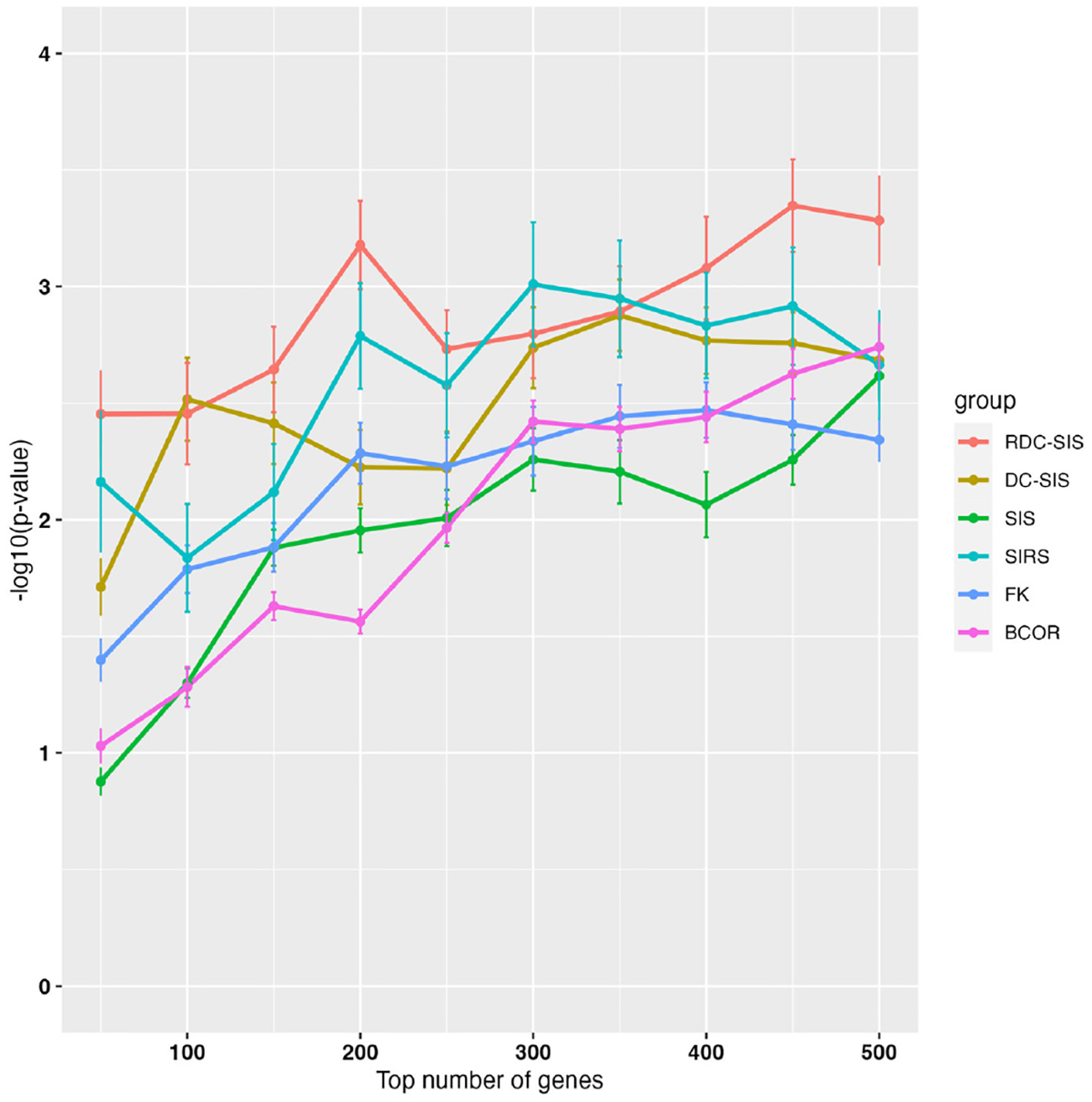
Plot of mean −log10 (p-value) with standard error from the Fisher’s exact tests of the 33 pathways in the intersection against the top number of genes used for each method.

An initial exploration of the data revealed that the expression of over half of the genes, along with the protein expression of MAPK1, exhibited a heavy-tailed distribution with kurtosis larger than 3, as illustrated in Figure 1. A total of 34 pathways were identified in the intersection of the top 200 pathways enriched by each method and used as the basis for comparison. These pathways mainly included interferon and other cytokine signaling, Ras and Rho signaling as well as cell cycle related pathways. Figure 2 showed that RDC outperformed all other methods in the overall enrichment of these ‘gold standard’ pathways and this trend remained consistent regardless of the number of top-ranked genes used. This result demonstrated that the ranking by our robust DC method surpassed other methods by prioritizing genes that are both more informative and biologically meaningful.

We then retained only the top d=⌊n/log(n)⌋=24 genes from each screening method and applied a second-stage regularization using Lasso. The tuning parameter for Lasso was chosen through cross-validation, employing the glmnet package (Friedman et al. 2010). Table 4 summarizes the MAE, adjusted $R^{2}$, and selected genes by each method. Among all methods, the final model selected by RDC-SIS+Lasso exhibited the largest adjusted $R^{2}$ value and the smallest MAE. Notably, there was minimal overlap among the genes selected by the various methods. The methods based on DC and RDC shared a significant proportion of matched genes (75%), with RDC uniquely identifying three genes: *ZFP*36*L*2, *COQ*2 and *IFIT*1. *ZFP*36*L*2 has been demonstrated to promote cancer cell aggressiveness in pancreatic ductal adenocarcinoma (Yonemori et al. 2017). *COQ*2 is involved in the synthesis of coenzyme Q10, the level of which in plasma has been associated with prostate cancer risk (Chai et al. 2011). *MAPK*1 gene was not featured in the top selections of any method, probably due to differences in translational rates and protein half-lives, among other factors (Edfors et al. 2016).

## Supplementary Material

Supplement

Additional supporting information can be found online in the Supporting Information section. **Data S1:** Supporting Information.

## Figures and Tables

**TABLE 1 |:** Scenario (I) results: Consider the same model as in the DC paper (Li et al. 2012), n=200,p=2000.

Models	Method	Minimum model size	${𝒫}_{1}$	${𝒫}_{a}$	$TP_{d_{1}}$	$TP_{d_{2}}$
Ia	RDC-SIS	9.5	1	0.88	3.9	3.92
	DC-SIS	9	1	0.88	3.9	3.94
	SIS	10	1	0.92	3.96	3.96
	SIRS	9	1	0.88	3.94	3.96
	FK	9.5	1	0.84	3.88	3.88
	NIS	11	1	0.9	3.9	3.92
	SC-SIS	10	1	0.66	3.78	3.8
	BCOR-SIS	9	1	0.8	3.82	3.82
Ib	RDC-SIS	13	1	0.88	3.94	3.94
	DC-SIS	13	1	0.9	3.94	3.98
	SIS	984.5	1	0.04	2.32	2.4
	SIRS	1014.5	1	0.02	2.1	2.18
	FK	30	1	0.52	3.42	3.5
	NIS	7	1	0.92	3.94	3.94
	SC-SIS	13	1	0.7	3.76	3.84
	BCOR-SIS	7.5	1	0.92	3.92	3.96
Ic	RDC-SIS	112	1	0.23	3.23	3.3
	DC-SIS	205.5	1	0.2	3.17	3.23
	SIS	516	1	0.1	2.77	2.87
	SIRS	125.5	1	0.33	3.37	3.4
	FK	132.5	1	0.33	3.27	3.33
	NIS	768	1	0.1	2.53	2.53
	SC-SIS	498.2	1	0.28	3.15	3.24
	BCOR-SIS	397.5	1	0.39	3.32	3.4
Id	RDC-SIS	20.5	1	0.5	3.63	3.77
	DC-SIS	87.5	0.97	0.3	3.1	3.23
	SIS	1440.5	0.43	0	0.7	0.77
	SIRS	1312	1	0	2	2
	FK	132	0.97	0.17	2.8	3.03
	NIS	1639.5	0.7	0	1.53	1.63
	SC-SIS	372.3	1	0.21	2.9	3.13
	BCOR-SIS	163.7	1	0.46	3.48	3.6
Ie	RDC-SIS	9	1	0.88	3.94	3.98
	DC-SIS	1838	0.56	0	0.78	0.88
	SIS	1546	0.32	0	0.52	0.76
	SIRS	8	1	0.88	3.92	3.94
	FK	9.5	1	0.78	3.9	3.9
	NIS	1907	0.4	0	0.6	0.76
	SC-SIS	9	1	0.82	3.86	3.88
	BCOR-SIS	9	1	0.72	3.8	3.86

**TABLE 2 |:** Scenario (II) results: n=100,p=2000,B=100.

Models	Method	Minimum model size	${𝒫}_{1}$	${𝒫}_{a}$	$TP_{d_{1}}$	$TP_{d_{2}}$
IIa	RDC-SIS	15.5	1	0.6	3.7	3.83
	DC-SIS	18	1	0.53	3.6	3.77
	SIS	157.5	0.93	0.1	2.83	3.07
	SIRS	38.5	1	0.47	3.53	3.67
	FK	32.5	1	0.37	3.5	3.6
	NIS	202	0.9	0.03	2.5	2.67
	SC-SIS	306.6	0.99	0.08	2.77	2.99
	BCOR-SIS	193	1	0.24	3.24	3.4
IIb	RDC-SIS	5	1	0.87	3.87	3.9
	DC-SIS	43.5	1	0.2	3.13	3.53
	Cor	117	1	0	1.97	2.63
	SIRS	16	1	0.53	3.67	3.83
	FK	14	1	0.7	3.8	3.83
	NIS	340	1	0.03	2	2.47
	SC-SIS	115.5	1	0.34	3.44	3.65
	BCOR-SIS	14.3	1	0.89	3.94	3.97

**TABLE 3 |:** Scenario (III) results: $y={\left(Y_{1},Y_{2},Y_{3}\right)}^{⊤}\sim x^{⊤}\beta ,n=100,p=2000,\rho_{X}=\rho_{Y}=0.5,B=100$.

Models	Method	Minimum model size	${𝒫}_{1}$	${𝒫}_{a}$	$TP_{d_{1}}$	$TP_{d_{2}}$
IIIa	RDC-SIS	4	1	1	2	2
	DC-SIS	5	0.97	0.87	1.93	1.93
	SIS	1137	0.07	0	0.1	0.2
	SIRS	1683.5	0.07	0	0.07	0.1
	FK	28.5	0.9	0.43	1.63	1.63
	NIS	2	1	0.87	1.9	1.97
	SC-SIS	389.2	0.41	0.11	0.71	0.86
	BCOR-SIS	351.7	0.55	0.17	0.9	1.07
IIIb	RDC-SIS	2	1	1	2	2
	DC-SIS	12.5	1	0.7	1.87	1.93
	SIS	41.5	1	0.43	1.53	1.67
	SIRS	3	1	0.83	1.83	1.87
	FK	2	1	1	2	2
	NIS	38	1	0.43	1.57	1.6
	SC-SIS	421.8	0.49	0.09	0.79	0.93
	BCOR-SIS	202.5	0.86	0.47	1.47	1.54

**TABLE 4 |:** Comparison of *MAE* (computed from leave-one-out cross-validation), adjusted $R^{2}$ and gene lists of the final models selected by different methods.

Methods	MAE	Adjusted $R^{2}$	Model size	Selected genes
RDC-SIS+Lasso	0.17	0.451	12	*CCNE1, CENPB, COQ2,HAPLN1,IFIT1,IKBKB, MX1,NUDT19,PDCD2L, SNTA1, USP18, ZFP36L2,ZNF204P*
DC-SIS+Lasso	0.2	0.436	12	*CCNE1, CENPB, CNIH3,HAPLN1,HOXD9,IKBKB, MX1,NUDT19,PDCD2L,PDCD5, SNTA1, USP18*
SIS+Lasso	0.17	0.394	8	*ATP6AP2,CCNE1,CENPB,CNIH3,FAM54A, HOXD9,IMPDH1,PDCD2L*
SIRS+Lasso	0.18	0.419	12	*C16orf80,CCNE1,CENPB,CNIH3, GPI,MX1, NUDT19,NUDT2, OAS2,PDCD2L, SNTA1, ZFP36L2*
FK+Lasso	0.18	0.419	10	*ASB14,C16orf80,CCNE1,COX4NB,E2F1,HAPLN1, IKBKB, PDCD2L, SNTA1, USP18*
BCOR+Lasso	0.21	0.315	5	*CCNE1,MX1,IKBKB,NDUFA7,ABCB8*

## Data Availability

The data that support the findings of this study are openly available in GitHub at https://github.com/kehongjie/RobustDC.
