# Supplementary material for: Robust Distance Correlation for Variable Screening
Source: Stat. Author manuscript; Available in PMC 2026 Aug 8. (PMC13450041; doi:10.1002/sta4.70094)
Supplement: Supplementary file 1 [file NIHMS2200005-supplement-Supplement.pdf]

Supplement to "Robust Distance Correlation for Variable Screening"

Tianzhou Ma*

Department of Epidemiology and Biostatistics, University of Maryland, MD, U.S.A.

Fan Yang*

Department of Statistics, University of Pittsburgh, PA, U.S.A.

Hongjie Ke

Department of Epidemiology and Biostatistics, University of Maryland, MD, U.S.A.

Zhao Ren

zren@pitt.edu

Department of Statistics, University of Pittsburgh, PA, U.S.A.

S1 Discussion

In this paper, we proposed a robust version of the distance correlation measure, termed "Robust DC", designed for variable screening in ultrahigh-dimensional data. This method addresses both model misspecification and tail robustness. It builds upon the original formulas for estimating distance correlation, substituting the summand with its truncated version to protect against heavy-tailedness. New phenomenon are observed in the optimal choice of robustification parameters, and our adopted approach leads to fewer tuning parameters, reducing the computation cost. Additionally, we developed a data-driven procedure to determine the optimal level of truncation

*Equal contribution.

by automatically tuning the related robustification parameters. Simulations under scenarios of various heavy-tailed distributions demonstrated the superiority of our screening method, which is based on robust DC, in prioritizing true active predictors when either the predictor or the outcome variables exhibit heavy tails. Applying this method to the heavy-tailed RNA-seq and RPPA data from the TCGA PAAD cohort, we found that Robust DC pinpointed more biologically informative pathways and identified unique genes associated with the progression mechanisms of pancreatic cancer.

Since their initial introduction by Fan and Lv (2008), sure screening methods based on different model-based or model-free measures have been proposed and shown their merits in variable selection for ultrahigh-dimensional regression and classification problems when coupled with regularization methods. Distance correlation-based sure screening is one such representative method that often sits as one of the top performers in various comparative analyses (Li et al., 2012). However, similar to many other model-free screening methods, this approach demonstrates robustness against model misspecification but fail to address the tail robustness. Heavy-tailedness is another significant characteristic of modern big data and its great impact on mean estimation has been reiterated in recent literature (Fan et al., 2017). Our method is one of the first sure screening approaches to tackle both model misspecification and tail robustness. The need to address heavy-tailedness is underscored by real-world data analyses, where several critical biomarkers are not selected by other methods due to the skewed distribution of RNA-seq data.

The data-driven tail truncation procedure for protecting against heavy-tailedness is applicable not only to the DC measure but can also be adapted for use with other measures, such as the Pearson correlation, the canonical correlation (He et al., 2021) and the partial correlation (Ke et al., 2022), etc. Moreover, since the marginal screening methods completely ignore the inter-feature dependency, one can also consider conducting a robust multivariate (or joint) screening that ranks each variable after controlling for other correlated variables (He et al., 2019). We demonstrated the efficacy of the proposed screening method on gene expression data, addressing the heavy-tailed nature prevalent in RNA-seq and microarray data. Furthermore, this method offers potential for extension to variable selection in other domains characterized by heavy-tailed distributions, such as neuroimaging or copy number variation data, etc.

S2 Additional notations

With slight abuse of notation in the proofs, for any random variable $X \in \mathbb{R}$, let $\|X\|_2 = [\mathbb{E}(X^2)]^{1/2}$ and $\|X\|_4 = [\mathbb{E}(X^4)]^{1/4}$ be the ℓ_2-norm and ℓ_4-norm, respectively. For simplicity, in the proofs we use $D(Y, X)$ and $\widehat{D}(Y, X)$ to represent $\text{dcov}^2(Y, X)$ and $\widehat{\text{rdcov}}^2(Y, X)$, respectively.

S3 Additional assumptions for RDC-REDS method

Assumption S1. *Recall $(\widetilde{Y}, \widetilde{X}_j)$ denotes an i.i.d. copy of (Y, X_j), and there exists a universal constant $\delta > 0$ and $\widetilde{C}$, such that, $[\mathbb{E}(|Y - \widetilde{Y}|^{4+\delta})]^{\frac{1}{4+\delta}} \leq \widetilde{C}$ and $\max_{1\leq j\leq p}[\mathbb{E}(|X_j - \widetilde{X}_j|^{4+\delta})]^{\frac{1}{4+\delta}} \leq \widetilde{C}$. Correspondingly, we have $(\log p)^{\frac{\delta+2}{4}}/n^{\frac{\delta}{4}} = o(1)$.*

Assumption S2. *For any $j \in \mathcal{D}^c$, the distance correlation between X_j and Y is negligible in the sense of that $\sup_{j\in\mathcal{D}^c} \sqrt{n}\omega_j = o(1)$.*

Assumption S3. *For any $j \in \mathcal{D}^c$, we define a four dimensional kernel vector,*

$$\mathbf{H} = \begin{pmatrix} |Y_1 - Y_2||X_{1j} - X_{2j}| - S_1^j \\ |Y_1 - Y_2| - S_{2,1}^j \\ |X_{1j} - X_{2j}| - S_{2,2}^j \\ \frac{1}{6}\sum_{k\neq\ell\neq m\in\{1,2,3\}} |Y_k - Y_\ell||X_{mj} - X_{\ell j}| - S_3^j \end{pmatrix}.$$

Subsequently, let $\mathbf{G} = \mathbb{E}(\mathbf{H}\big|(Y_1, X_{1j}))$, and denote $\mathbf{V} = \mathbb{E}(\mathbf{G}\mathbf{G}^\top)$. Assume that there exists a constant $0 < \epsilon < 1$, such that $\epsilon < \lambda_{min}(\mathbf{V}) < \lambda_{max}(\mathbf{V}) < 1/\epsilon$, where $\lambda_{min}(\cdot)$ and $\lambda_{max}(\cdot)$ denote the smallest and largest eigenvalues respectively and $\|\mathbf{H}\|_2 = \sqrt{\mathbf{H}^\top\mathbf{H}}$.

Assumption S4. *For the REDS statistics W_j's, $j = 1, \ldots, p$ defined in Algorithm 2, there exist constants $c_\gamma > 0$ and $\gamma < 2$ such that for any $t \in \mathbb{R}$,*

$$\text{Var}\left(\sum_{j\in\mathcal{D}^c} \mathbb{1}\{W_j > t\}\right) \leq c_\gamma p_0^\gamma,$$

where $p_0 = |\mathcal{D}^c| = p - s$.

S4 Proofs and key lemmas

The Lemma S1 below offers a slightly different result from the one presented in Arcones (1995), which assumes the necessity of a symmetric kernel function. However, we find that symmetry in the kernel is not required. Our lemma can be proven by leveraging the representation of U-statistics as elucidated in Hoeffding (1963), coupled with a straightforward application of the Bernstein inequality. For brevity, we do not include the detailed proof of this lemma.

Lemma S1. *For i.i.d. random variables W_i, $i = 1, \ldots, n$, let $h(W_{i_1}, \ldots, W_{i_m})$ be the kernel function of order m, such that $\mathbb{E}(h(W_{i_1}, \ldots, W_{i_m})) = 0$, $\mathrm{Var}(h(W_{i_1}, \ldots, W_{i_m})) = \sigma^2$ and $|h(W_{i_1}, \ldots, W_{i_m})| \leq b$ almost surely. Define the U-statistic as follows,*

$$U_n(h) = \frac{1}{n(n-1)\cdots(n-m+1)} \sum_{(i_1,\ldots,i_m)\in P_m^n} h(W_{i_1}, \ldots, W_{i_m}),$$

where we denote $P_m^n = \{(i_1, \ldots, i_m) : 1 \leq i_1 \neq \ldots \neq i_m \leq n\}$. Then we have that,

$$\mathbb{P}(|U_n(h)| \geq y) \leq 2\exp\{-\frac{\lfloor \frac{n}{m} \rfloor y^2}{2\sigma^2 + \frac{2}{3}by}\}.$$

The following two lemmas are used in the theoretical analysis of FDR control for the procedure proposed in Algorithm 2. The proof of Lemma S2 will be provided later.

Lemma S2. *Under Assumptions 2, S1 and S2, for any $j \in \mathcal{D}^c$, we have*

$$|(S_1^j + S_2^j - 2S_3^j) - (S_1^{*j} + S_2^{*j} - 2S_3^{*j})| \leq K'(\log p/n)^{(\delta+2)/4},$$

where K' is some universal constant and δ is the constant in Assumption S1.

Lemma S3 (Theorem 1.16 in Götze (1987))**.** *For i.i.d. random variables X_i's, $i = 1, \ldots, n$ and a vector-valued kernel $\mathbf{H}(x, y) = (H_1(x, y), \ldots, H_k(x, y))^\top$, a k-variate U-statistic of degree two is defined as*

$$\mathbf{U}_n = \frac{2}{n(n-1)} \sum_{1\leq i<j\leq n} \mathbf{H}(X_i, X_j).$$

Furthermore, define $\mathbf{U}_n^* = \frac{1}{2}\sqrt{n}\mathbf{U}_n$, $\mathbf{G} = \mathbb{E}(\mathbf{H}(X_1, X_2)|X_1)$, and $\mathbf{V} = \mathbb{E}(\mathbf{G}\mathbf{G}^\top)$. Assume that

$\mathbb{E}(\mathbf{H}(X_1, X_2)) = 0$, $\mathbb{E}(\|\mathbf{H}(X_1, X_2)\|_2^3) \le A < \infty$, *and the smallest eigenvalue of* $\mathbf{V}$ *is larger than* $\epsilon > 0$.

Then, for any $B > 0$, *there exist constants* $d_1, d_2 > 0$ *depending on* ϵ, A, B, *such that*

$$\sup_{E \in L_{B,v_n}} |\mathbb{P}(\mathbf{U}_n^* \in E) - \int_E \phi_{0,V}(x) d^k x| \le d_2/\sqrt{n},$$

where $v_n = d_1/\sqrt{n}$, $L_{B,v_n} = \{E : \int_{(\partial E)^f} \phi_{0,V}(x) d^k x \le Bf + v_n, \text{for any } f > 0\}$, $(\partial E)^f = \{x : \exists y \in \partial E, \|x - y\|_2 \le f\}$, *and* $\phi_{0,V}(x)$ *is the probability function of normal distribution with mean 0 and covariance matrix* $\mathbf{V}$.

S4.1 Proof of Proposition 1

The upper bound for robustification bias can be found in the proof of Theorem 1 (see, for example, Equation (3) for the upper bound of $S_1^j - S_1^{*j}$). Therefore, we do not provide a separate proof for Proposition 1.

S4.2 Proof of Theorem 1

Proof. To demonstrate the consistency of $\hat{\omega}_j^\tau = \frac{\widehat{D}(Y, X_j)}{\sqrt{\widehat{D}(Y,Y)\widehat{D}(X_j,X_j)}}$, we firstly deal with the numerator $\widehat{D}(Y, X_j) = \widehat{S}_1^{\tau_j,\tau_y} + \widehat{S}_2^{\tau_j',\tau_y'} - 2\widehat{S}_3^{\tau_j,\tau_y}$. Recall that $(\widetilde{Y}, \widetilde{X}_j)$ is an independent copy of (Y, X_j), and we have $\tau_y = \|Y - \widetilde{Y}\|_4 (n/t)^{1/4}$ and $\tau_j = \|X_j - \widetilde{X}_j\|_4 (n/t)^{1/4}$ with $t = o(n)$. Throughout the proof, we just assume that there exists a sufficiently small constant $0 < c' < 1$ such that $t < c'n$.

Recall that $\widehat{S}_1^{\tau_j,\tau_y} = \frac{1}{n^2}\sum_{k=1}^n \sum_{\ell=1}^n |\psi_{\tau_y}(Y_k - Y_\ell)||\psi_{\tau_j}(X_{kj} - X_{\ell j})|$. Furthermore, we define $\widehat{S}_1^{*\tau_j,\tau_y} = \frac{1}{n(n-1)} \sum_{(k,\ell)\in P_2^n} \psi_{\tau_y}(|Y_k - Y_\ell|)\psi_{\tau_j}(|X_{kj} - X_{\ell j}|)$. Subsequently, define $S_1^j = \mathbb{E}(|Y - \widetilde{Y}||X_j - \widetilde{X}_j|)$ and $S_1^{*j} = \mathbb{E}(\psi_{\tau_y}(|Y - \widetilde{Y}|)\psi_{\tau_j}(|X_j - \widetilde{X}_j|))$. To bound the bias term, that is, the difference between S_1^j and S_1^{*j}, we denote $\alpha_j = \psi_{\tau_j}(|X_j - \widetilde{X}_j|)/|X_j - \widetilde{X}_j|$ and $\alpha_y = \psi_{\tau_y}(|Y - \widetilde{Y}|)/|Y - \widetilde{Y}|$.

Note that whenever $|Y - \widetilde{Y}| \geq \tau_y$, we get that

$$\frac{|Y - \widetilde{Y}| - \tau_y}{|Y - \widetilde{Y}|} \Bigg/ \frac{|Y - \widetilde{Y}|^2}{\tau_y^2} = \frac{\tau_y^2(|Y - \widetilde{Y}| - \tau_y)}{|Y - \widetilde{Y}|^3}$$
$$\leq \max_{r \geq 1} \frac{r-1}{r^3}$$
$$= \frac{4}{27}.$$

Consequently, we have,

$$\begin{aligned} 1 - \alpha_y &= \frac{|Y - \widetilde{Y}| - \psi_{\tau_y}(|Y - \widetilde{Y}|)}{|Y - \widetilde{Y}|} \\ &= \mathbb{1}\left\{|Y - \widetilde{Y}| \geq \tau_y\right\} \frac{|Y - \widetilde{Y}| - \tau_y}{|Y - \widetilde{Y}|} \\ &\leq \frac{4}{27} \frac{|Y - \widetilde{Y}|^2}{\tau_y^2}. \end{aligned} \tag{1}$$

Analogously, we can also show that,

$$1 - \alpha_j \leq \frac{4}{27} \frac{|X_j - \widetilde{X}_j|^2}{\tau_j^2}. \tag{2}$$

Therefore, we obtain that,

$$\begin{aligned} S_1^j - S_1^{*j} &= \mathbb{E}\left((1 - \alpha_j \alpha_y)|Y - \widetilde{Y}||X_j - \widetilde{X}_j|\right) \\ &\leq \mathbb{E}\left((1 - \alpha_y)|Y - \widetilde{Y}||X_j - \widetilde{X}_j|\right) + \mathbb{E}\left(\alpha_y(1 - \alpha_j)|Y - \widetilde{Y}||X_j - \widetilde{X}_j|\right) \\ &\leq \mathbb{E}\left((1 - \alpha_y)|Y - \widetilde{Y}||X_j - \widetilde{X}_j|\right) + \mathbb{E}\left((1 - \alpha_j)|Y - \widetilde{Y}||X_j - \widetilde{X}_j|\right) \\ &\leq \frac{4}{27}\left(\frac{1}{\tau_y^2}\mathbb{E}(|Y - \widetilde{Y}|^3|X_j - \widetilde{X}_j|) + \frac{1}{\tau_j^2}\mathbb{E}(|Y - \widetilde{Y}||X_j - \widetilde{X}_j|^3)\right), \end{aligned} \tag{3}$$

where the last inequality follows from (1) and (2).

By Hölder's inequality and Cauchy–Schwarz inequality, we have that,

$$\begin{aligned} \mathbb{E}(|Y - \widetilde{Y}|^3|X_j - \widetilde{X}_j|) &\leq \left(\mathbb{E}(|Y - \widetilde{Y}|^4)\right)^{3/4} \left(\mathbb{E}(|X_j - \widetilde{X}_j|^4)\right)^{1/4} \\ &= \|Y - \widetilde{Y}\|_4^3 \|X_j - \widetilde{X}_j\|_4. \end{aligned} \tag{4}$$

Similarly, we also have,

$$\mathbb{E}(|Y - \widetilde{Y}||X_j - \widetilde{X}_j|^3) \le \|Y - \widetilde{Y}\|_4 \|X_j - \widetilde{X}_j\|_4^3. \tag{5}$$

Then, by (3), (4), (5) and Assumption 1, we obtain that,

$$S_1^j - S_1^{*j} \le \frac{8}{27}\|Y - \widetilde{Y}\|_4 \|X_j - \widetilde{X}_j\|_4 (\frac{t}{n})^{1/2} \le \frac{8}{27}\widetilde{C}^2 (\frac{t}{n})^{1/2}. \tag{6}$$

It is worth noting that both S_1^j and S_1^{*j} are bounded. More specifically, we have that,

$$\begin{aligned} S_1^{*j} &\le S_1^j \\ &\le \frac{1}{2}(\mathbb{E}(|Y - \widetilde{Y}|^2) + \mathbb{E}(|X_j - \widetilde{X}_j|^2)) \\ &\le \frac{1}{2}(\|Y - \widetilde{Y}\|_4^2 + \|X_j - \widetilde{X}_j\|_4^2) \\ &\le \widetilde{C}^2, \end{aligned} \tag{7}$$

where the second inequality follows from the fact that $2ab \le a^2 + b^2$, while the third inequality follows from the fact that ℓ_2-norm is no larger than ℓ_4-norm.

We define the kernel function $h_1(Y_k, X_{kj}; Y_\ell, X_{\ell j}) = \psi_{\tau_y}(|Y_k - Y_\ell|)\psi_{\tau_j}(|X_{kj} - X_{\ell j}|) - S_1^{*j}$. By (7) it is evident to see that $-\widetilde{C}^2 \le h_1 \le \tau_y \tau_j$. When n is large enough, we have $\tau_y \tau_j > \widetilde{C}^2$. Consequently, we can conclude that $|h_1| \le \tau_y \tau_j$ for large n. Note that $\mathbb{E}(h_1) = 0$, and we have that,

$$\begin{aligned} \mathrm{Var}(h_1) &\le \mathbb{E}(|Y - \widetilde{Y}|^2 |X_j - \widetilde{X}_j|^2) \\ &\le [\mathbb{E}(|Y - \widetilde{Y}|^4)]^{1/2} [\mathbb{E}(|X_j - \widetilde{X}_j|^4)]^{1/2} \\ &= \|Y - \widetilde{Y}\|_4^2 \|X_j - \widetilde{X}_j\|_4^2. \end{aligned} \tag{8}$$

Therefore, by Lemma S1 and (8), we can get,

$$\mathbb{P}\left(|\widehat{S}_1^{*\tau_j,\tau_y} - S_1^{*j}| \ge y\right) \le 2\exp\left\{-\frac{\lfloor \frac{n}{2} \rfloor y^2}{2\|Y - \widetilde{Y}\|_4^2 \|X_j - \widetilde{X}_j\|_4^2 + \frac{2}{3}\tau_y \tau_j y}\right\}. \tag{9}$$

We pick $y = 3\|Y - \widetilde{Y}\|_4 \|X_j - \widetilde{X}_j\|_4 \sqrt{\frac{t}{n}} \leq 3\widetilde{C}^2 \sqrt{\frac{t}{n}}$. By (9) and the fact that $9\lfloor \frac{n}{2} \rfloor \frac{1}{n} > 4$ for all $n \geq 9$, we can obtain that,

$$\mathbb{P}\left(|\widehat{S}_1^{*\tau_j, \tau_y} - S_1^{*j}| \geq 3\widetilde{C}^2 \sqrt{\frac{t}{n}} \right) \leq 2\exp\{-t\}. \tag{10}$$

Notice that,

$$\begin{aligned} |\widehat{S}_1^{\tau_j, \tau_y} - S_1^j| &= |\frac{n-1}{n}\widehat{S}_1^{*\tau_j, \tau_y} - \frac{n-1}{n}S_1^{*j} + S_1^{*j} - S_1^j - \frac{1}{n}S_1^{*j}| \\ &\leq \frac{n-1}{n}|\widehat{S}_1^{*\tau_j, \tau_y} - S_1^{*j}| + |S_1^{*j} - S_1^j| + \frac{1}{n}S_1^{*j}. \end{aligned} \tag{11}$$

By (6), (7), and Assumption 1, there exists a universal constant C_1, such that ,

$$\frac{n}{n-1}(C_1\sqrt{\frac{t}{n}} - |S_1^{*j} - S_1^j| - \frac{1}{n}S_1^{*j}) \geq 3\widetilde{C}^2 \sqrt{\frac{t}{n}}. \tag{12}$$

Therefore, by (10), (11), and (12), we can conclude that,

$$\begin{aligned} \mathbb{P}\left(|\widehat{S}_1^{\tau_j, \tau_y} - S_1^j| \geq C_1 \sqrt{\frac{t}{n}} \right) &\leq \mathbb{P}\left(|\widehat{S}_1^{*\tau_j, \tau_y} - S_1^{*j}| \geq \frac{n}{n-1}(C_1\sqrt{\frac{t}{n}} - |S_1^{*j} - S_1^j| - \frac{1}{n}S_1^{*j}) \right) \\ &\leq \mathbb{P}\left(|\widehat{S}_1^{*\tau_j, \tau_y} - S_1^{*j}| \geq 3\widetilde{C}^2 \sqrt{\frac{t}{n}} \right) \\ &\leq 2\exp\{-t\}. \end{aligned} \tag{13}$$

Recall that $\widehat{S}_2^{\tau_j', \tau_y'} = \left(\frac{1}{n^2} \sum_{k=1}^n \sum_{\ell=1}^n |\psi_{\tau_j'}(X_{kj} - X_{\ell j})| \right) \left(\frac{1}{n^2} \sum_{k=1}^n \sum_{\ell=1}^n |\psi_{\tau_y'}(Y_k - Y_\ell)| \right)$, where

$\tau_y' = \|Y - \widetilde{Y}\|_2 (n/t)^{1/2}$ and $\tau_j' = \|X_j - \widetilde{X}_j\|_2 (n/t)^{1/2}$. We further define the following notations,

$$
\begin{aligned}
\widehat{S}_{2,1}^{\tau_y'} &= \frac{1}{n^2} \sum_{k=1}^{n} \sum_{\ell=1}^{n} |\psi_{\tau_y'}(Y_k - Y_\ell)|, \\
\widehat{S}_{2,1}^{*\tau_y'} &= \frac{1}{n(n-1)} \sum_{(k,\ell)\in P_2^n} \psi_{\tau_y'}(|Y_k - Y_\ell|), \\
S_{2,1}^{j} &= \mathbb{E}(|Y - \widetilde{Y}|), \\
S_{2,1}^{*j} &= \mathbb{E}(\psi_{\tau_y'}(|Y - \widetilde{Y}|)), \\
\widehat{S}_{2,2}^{\tau_j'} &= \frac{1}{n^2} \sum_{k=1}^{n} \sum_{\ell=1}^{n} |\psi_{\tau_j'}(X_{kj} - X_{\ell j})|, \\
\widehat{S}_{2,2}^{*\tau_j'} &= \frac{1}{n(n-1)} \sum_{(k,\ell)\in P_2^n} \psi_{\tau_j'}(|X_{kj} - X_{\ell j}|), \\
S_{2,2}^{j} &= \mathbb{E}(|X_j - \widetilde{X}_j|), \\
S_{2,2}^{*j} &= \mathbb{E}(\psi_{\tau_j'}(|X_j - \widetilde{X}_j|)).
\end{aligned}
$$

Denote $\beta_y = \psi_{\tau_y'}(|Y - \widetilde{Y}|)/|Y - \widetilde{Y}|$. Note that if $|Y - \widetilde{Y}| \geq \tau_y'$, we then have,

$$
\begin{aligned}
\frac{|Y - \widetilde{Y}| - \tau_y'}{|Y - \widetilde{Y}|} \Bigg/ \frac{|Y - \widetilde{Y}|}{\tau_y'} &= \frac{\tau_y'(|Y - \widetilde{Y}| - \tau_y')}{|Y - \widetilde{Y}|^2} \\
&\leq \max_{r\geq 1} \frac{r-1}{r^2} \\
&= \frac{1}{4}.
\end{aligned}
$$

Consequently, we can obtain that,

$$
\begin{aligned}
S_{2,1}^{j} - S_{2,1}^{*j} &= \mathbb{E}\left((1 - \beta_y)|Y - \widetilde{Y}|\right) \\
&= \mathbb{E}\left(\mathbb{1}\left\{|Y - \widetilde{Y}| \geq \tau_y'\right\} \frac{|Y - \widetilde{Y}| - \tau_y'}{|Y - \widetilde{Y}|} \times |Y - \widetilde{Y}|\right) \\
&\leq \frac{1}{4\tau_y'} \mathbb{E}(|Y - \widetilde{Y}|^2) \\
&= \frac{1}{4} \|Y - \widetilde{Y}\|_2 \sqrt{\frac{t}{n}} \\
&\leq \frac{\widetilde{C}}{4} \sqrt{\frac{t}{n}}. \qquad (14)
\end{aligned}
$$

It is evident to see that both $S^j_{2,1}$ and $S^{*j}_{2,1}$ are bounded. To be more specific, we have

$$S^{*j}_{2,1} \leq S^j_{2,1} \leq \|Y - \widetilde{Y}\|_4 \leq \widetilde{C}. \tag{15}$$

Define $h_{2,1}(Y_k, Y_\ell) = \psi_{\tau'_y}(|Y_k - Y_\ell|) - S^{*j}_{2,1}$. Then, by (15) we can show that $|h_{2,1}| \leq \tau'_y$, when n is sufficiently large. Furthermore, we have,

$$\mathrm{Var}(h_{2,1}) \leq \mathbb{E}(|Y - \widetilde{Y}|^2) \leq \|Y - \widetilde{Y}\|_2^2. \tag{16}$$

Consequently, by Lemma S1 and (16), we can obtain,

$$\mathbb{P}\left(|\widehat{S}^{*\tau'_y}_{2,1} - S^{*j}_{2,1}| \geq y\right) \leq 2\exp\left\{-\frac{\lfloor \frac{n}{2} \rfloor y^2}{2\|Y - \widetilde{Y}\|_2^2 + \frac{2}{3}\tau'_y y}\right\}.$$

Similarly, by picking $y = 3\|Y - \widetilde{Y}\|_2\sqrt{\frac{t}{n}}$, we have

$$\mathbb{P}\left(|\widehat{S}^{*\tau'_y}_{2,1} - S^{*j}_{2,1}| \geq 3\widetilde{C}\sqrt{\frac{t}{n}}\right) \leq 2\exp\{-t\}.$$

Note that,

$$\begin{aligned}|\widehat{S}^{\tau'_y}_{2,1} - S^j_{2,1}| &= |\frac{n-1}{n}(\widehat{S}^{*\tau'_y}_{2,1} - S^{*j}_{2,1}) + (S^{*j}_{2,1} - S^j_{2,1}) - \frac{1}{n}S^{*j}_{2,1}| \\ &\leq \frac{n-1}{n}|\widehat{S}^{*\tau'_y}_{2,1} - S^{*j}_{2,1}| + |S^{*j}_{2,1} - S^j_{2,1}| + \frac{1}{n}S^{*j}_{2,1}.\end{aligned}$$

By (14) and (15), there exists a universal constant $C_{2,1}$, such that,

$$\frac{n}{n-1}(C_{2,1}\sqrt{\frac{t}{n}} - |S^{*j}_{2,1} - S^j_{2,1}| - \frac{1}{n}S^{*j}_{2,1}) \geq 3\widetilde{C}\sqrt{\frac{t}{n}}.$$

Consequently, following similar argument to that in (13), we obtain,

$$\mathbb{P}\left(|\widehat{S}^{\tau'_y}_{2,1} - S^j_{2,1}| \geq C_{2,1}\sqrt{\frac{t}{n}}\right) \leq 2\exp\{-t\}.$$

Analogously, there exists a universal constant $C_{2,2}$, such that,

$$\mathbb{P}\left(|\widehat{S}_{2,2}^{\tau_j'} - S_{2,2}^j| \geq C_{2,2}\sqrt{\frac{t}{n}}\right) \leq 2\exp\{-t\}. \tag{17}$$

Note that,

$$\begin{aligned}\widehat{S}_2^{\tau_j',\tau_y'} - S_2^j &= \widehat{S}_{2,1}^{\tau_y'}\widehat{S}_{2,2}^{\tau_j'} - S_{2,1}^j S_{2,2}^j \\ &= (\widehat{S}_{2,1}^{\tau_y'} - S_{2,1}^j)(\widehat{S}_{2,2}^{\tau_j'} - S_{2,2}^j) + (\widehat{S}_{2,1}^{\tau_y'} - S_{2,1}^j)S_{2,2}^j + (\widehat{S}_{2,2}^{\tau_j'} - S_{2,2}^j)S_{2,1}^j.\end{aligned}$$

Since both $S_{2,1}^j$ and $S_{2,2}^j$ are bounded, there exists a universal constant C_2, such that we have $\min\{\frac{C_2}{3}, \frac{C_2}{3S_{2,1}^j}, \frac{C_2}{3S_{2,2}^j}\} \geq \max\{C_{2,1}, C_{2,2}\}$. With the assumption that $t < c'n$ for a sufficiently small constant $0 < c' < 1$, we then have $\frac{C_2}{3}\sqrt{\frac{t}{n}} < 1$. Therefore, we conclude that,

$$\begin{aligned}\mathbb{P}\left(|\widehat{S}_2^{\tau_j',\tau_y'} - S_2^j| \geq C_2\sqrt{\frac{t}{n}}\right) &\leq \mathbb{P}\left(|\widehat{S}_{2,1}^{\tau_y'} - S_{2,1}^j| \geq (\frac{C_2}{3})^{1/2}(\frac{t}{n})^{1/4}\right) + \mathbb{P}\left(|\widehat{S}_{2,2}^{\tau_j'} - S_{2,2}^j| \geq (\frac{C_2}{3})^{1/2}(\frac{t}{n})^{1/4}\right) \\ &\quad + \mathbb{P}\left(|(\widehat{S}_{2,1}^{\tau_y'} - S_{2,1}^j)S_{2,2}^j| \geq \frac{C_2}{3}\sqrt{\frac{t}{n}}\right) + \mathbb{P}\left(|(\widehat{S}_{2,2}^{\tau_j'} - S_{2,2}^j)S_{2,1}^j| \geq \frac{C_2}{3}\sqrt{\frac{t}{n}}\right) \\ &\leq \mathbb{P}\left(|\widehat{S}_{2,1}^{\tau_y'} - S_{2,1}^j| \geq \frac{C_2}{3}\sqrt{\frac{t}{n}}\right) + \mathbb{P}\left(|\widehat{S}_{2,2}^{\tau_j'} - S_{2,2}^j| \geq \frac{C_2}{3}\sqrt{\frac{t}{n}}\right) \\ &\quad + \mathbb{P}\left(|\widehat{S}_{2,1}^{\tau_y'} - S_{2,1}^j| \geq \frac{C_2}{3S_{2,2}^j}\sqrt{\frac{t}{n}}\right) + \mathbb{P}\left(|\widehat{S}_{2,2}^{\tau_j'} - S_{2,2}^j| \geq \frac{C_2}{3S_{2,1}^j}\sqrt{\frac{t}{n}}\right) \\ &\leq 2\mathbb{P}\left(|\widehat{S}_{2,1}^{\tau_y'} - S_{2,1}^j| \geq C_{2,1}\sqrt{\frac{t}{n}}\right) + 2\mathbb{P}\left(|\widehat{S}_{2,2}^{\tau_j'} - S_{2,2}^j| \geq C_{2,2}\sqrt{\frac{t}{n}}\right) \\ &\leq 8\exp\{-t\}. \end{aligned} \tag{18}$$

Recall that $\widehat{S}_3^{\tau_j,\tau_y} = \frac{1}{n^3}\sum_{k=1}^n\sum_{\ell=1}^n\sum_{m=1}^n |\psi_{\tau_y}(Y_k - Y_\ell)||\psi_{\tau_j}(X_{mj} - X_{\ell j})|$. Accordingly, we define $\widehat{S}_3^{*\tau_j,\tau_y} = \sum_{(k,\ell,m)\in P_3^n} \psi_{\tau_y}(|Y_k - Y_\ell|)\psi_{\tau_j}(|X_{mj} - X_{\ell j}|)$. Let $(\widetilde{Y}, \widetilde{X}_j)$ and $(\check{Y}, \check{X}_j)$ be two i.i.d. copies of (Y, X_j). Subsequently, define $S_3^j = \mathbb{E}(|\widetilde{Y} - Y||\check{X}_j - X_j|)$ and $S_3^{*j} = \mathbb{E}(\psi_{\tau_y}(|\widetilde{Y} - Y|)\psi_{\tau_j}(|\check{X}_j - X_j|))$. Analogous to the argument in (6) and (7), we can show that,

$$S_3^j - S_3^{*j} \leq \frac{8}{27}\widetilde{C}^2(\frac{t}{n})^{1/2}, \tag{19}$$

and

$$S_3^{*j} \le S_3^j \le \widetilde{C}^2. \tag{20}$$

Define $h_3(Y_k, X_{kj}; Y_\ell, X_{\ell j}; Y_m, X_{mj}) = \psi_{\tau_y}(|Y_k - Y_\ell|)\psi_{\tau_j}(|X_{mj} - X_{\ell j}|) - S_3^{*j}$. Then we can show that $|h_3| \le \tau_j\tau_y$ for large n, and $\mathrm{Var}(h_3) \le \|Y - \widetilde{Y}\|_4^2\|X_j - \widetilde{X}_j\|_4^2$. Therefore, by Lemma S1, we obtain that,

$$\mathbb{P}\left(|\widehat{S}_3^{*\tau_j,\tau_y} - S_3^{*j}| \ge y\right) \le 2\exp\left\{-\frac{\lfloor \frac{n}{3} \rfloor y^2}{2\|Y - \widetilde{Y}\|_4^2\|X_j - \widetilde{X}_j\|_4^2 + \frac{2}{3}\tau_y\tau_j y}\right\}.$$

Similar to the argument for (10), by picking $y = 4\|Y - \widetilde{Y}\|_4\|X_j - \widetilde{X}_j\|_4\sqrt{\frac{t}{n}}$, we can then conclude,

$$\mathbb{P}\left(|\widehat{S}_3^{*\tau_j,\tau_y} - S_3^{*j}| \ge 4\widetilde{C}^2\sqrt{\frac{t}{n}}\right) \le 2\exp\{-t\}.$$

Note that,

$$\begin{aligned}
|\widehat{S}_3^{\tau_j,\tau_y} - S_3^j| &= |\frac{(n-1)(n-2)}{n^2}(\widehat{S}_3^{*\tau_j,\tau_y} + \frac{1}{n-2}\widehat{S}_1^{*\tau_j,\tau_y}) - S_3^j| \\
&\le \frac{(n-1)(n-2)}{n^2}|\widehat{S}_3^{*\tau_j,\tau_y} - S_3^{*j}| + \frac{(n-1)(n-2)}{n^2}|S_3^{*j} - S_3^j| \\
&+ \frac{3n-2}{n^2}S_3^j + \frac{n-1}{n^2}|\widehat{S}_1^{*\tau_j,\tau_y} - S_1^{*j}| + \frac{n-1}{n^2}S_1^{*j}.
\end{aligned} \tag{21}$$

By (7), (19), (20), and Assumption 1, there exists a universal constant C_3, such that,

$$\frac{C_3}{5}\sqrt{\frac{t}{n}} \ge \max\left\{\frac{(n-1)(n-2)}{n^2}|S_3^{*j} - S_3^j|, \frac{3n-2}{n^2}S_3^j, \frac{n-1}{n^2}S_1^{*j}\right\},$$

and

$$\frac{C_3}{5} \times \frac{n^2}{(n-1)(n-2)} \ge 4\widetilde{C}^2.$$

Consequently, by (21), we conclude that,

$$\mathbb{P}\left(|\widehat{S}_3^{\tau_j,\tau_y} - S_3^j| \geq C_3\sqrt{\frac{t}{n}}\right) \leq \mathbb{P}\left(|\widehat{S}_3^{*\tau_j,\tau_y} - S_3^{*j}| \geq \frac{C_3}{5} \times \frac{n^2}{(n-1)(n-2)}\sqrt{\frac{t}{n}}\right)$$
$$+ \mathbb{P}\left(|\widehat{S}_1^{*\tau_j,\tau_y} - S_1^{*j}| \geq \frac{C_3}{5} \times \frac{n^2}{n-1}\sqrt{\frac{t}{n}}\right)$$
$$\leq 4\exp\{-t\}. \tag{22}$$

Recall that $\widehat{D}(Y, X_j) = \widehat{S}_1^{\tau_j,\tau_y} + \widehat{S}_2^{\tau_j',\tau_y'} - 2\widehat{S}_3^{\tau_j,\tau_y}$. Let $C_u = 4\max\{C_1, C_2, C_3\}$. Then, by (13), (18), and (22), we conclude that,

$$\mathbb{P}\left(|\widehat{D}(Y, X_j) - D(Y, X_j)| \geq C_u\sqrt{\frac{t}{n}}\right) \leq \mathbb{P}\left(|\widehat{S}_1^{\tau_j,\tau_y} - S_1^j| \geq \frac{C_u}{4}\sqrt{\frac{t}{n}}\right)$$
$$+ \mathbb{P}\left(|\widehat{S}_2^{\tau_j',\tau_y'} - S_2^j| \geq \frac{C_u}{4}\sqrt{\frac{t}{n}}\right) + \mathbb{P}\left(|\widehat{S}_3^{\tau_j,\tau_y} - S_3^j| \geq \frac{C_u}{4}\sqrt{\frac{t}{n}}\right)$$
$$\leq 14\exp\{-t\}. \tag{23}$$

With a similar argument, we can also show that,

$$\mathbb{P}\left(|\widehat{D}(Y, Y) - D(Y, Y)| \geq C_u\sqrt{\frac{t}{n}}\right) \leq 14\exp\{-t\}, \tag{24}$$

and

$$\mathbb{P}\left(|\widehat{D}(X_j, X_j) - D(X_j, X_j)| \geq C_u\sqrt{\frac{t}{n}}\right) \leq 14\exp\{-t\}. \tag{25}$$

Note that,

$$|\hat{\omega}_j^\tau - \omega_j| = \left|\frac{\widehat{D}(Y, X_j)}{\sqrt{\widehat{D}(Y, Y)\widehat{D}(X_j, X_j)}} - \frac{D(Y, X_j)}{\sqrt{D(Y, Y)D(X_j, X_j)}}\right|$$
$$\leq \left|(\widehat{D}(Y, X_j) - D(Y, X_j))(\frac{1}{\sqrt{\widehat{D}(Y, Y)\widehat{D}(X_j, X_j)}} - \frac{1}{\sqrt{D(Y, Y)D(X_j, X_j)}})\right|$$
$$+ \left|(\widehat{D}(Y, X_j) - D(Y, X_j))\frac{1}{\sqrt{D(Y, Y)D(X_j, X_j)}}\right|$$
$$+ \left|D(Y, X_j)(\frac{1}{\sqrt{\widehat{D}(Y, Y)\widehat{D}(X_j, X_j)}} - \frac{1}{\sqrt{D(Y, Y)D(X_j, X_j)}})\right|. \tag{26}$$

By Assumption 1, we have that $D(Y,Y)$ and $D(X_j,X_j)$ are both upper bounded by some universal constant. Therefore, following similar argument to that in (18), with (24) and (25) we can conclude that there exists a universal constant C_d, such that,

$$\mathbb{P}\left(|\widehat{D}(Y,Y)\widehat{D}(X_j,X_j) - D(Y,Y)D(X_j,X_j)| \geq C_d\sqrt{\frac{t}{n}}\right) \leq 56\exp\{-t\}. \tag{27}$$

By Assumption 2, we have that $D(Y,Y)D(X_j,X_j) \geq \widetilde{c}^2$. Subsequently, by mean value theorem, there exists x_0 between $D(Y,Y)D(X_j,X_j)$ and $\widehat{D}(Y,Y)\widehat{D}(X_j,X_j)$, such that

$$|\frac{1}{\sqrt{\widehat{D}(Y,Y)\widehat{D}(X_j,X_j)}} - \frac{1}{\sqrt{D(Y,Y)D(X_j,X_j)}}| = \frac{1}{2x_0^{3/2}}|\widehat{D}(Y,Y)\widehat{D}(X_j,X_j) - D(Y,Y)D(X_j,X_j)|. \tag{28}$$

Define the following event,

$$\mathcal{A} = \left\{|\widehat{D}(Y,Y)\widehat{D}(X_j,X_j) - D(Y,Y)D(X_j,X_j)| \leq C_d\sqrt{\frac{t}{n}}\right\}.$$

Since $t < c'n$ for sufficiently small constant $0 < c' < 1$, we can get that $\widetilde{c}^2 - C_d\sqrt{\frac{t}{n}} \geq \frac{\widetilde{c}^2}{2}$. Therefore, conditional on event $\mathcal{A}$, we have $x_0 \geq \frac{\widetilde{c}^2}{2}$. Define another event as follows,

$$\mathcal{B} = \left\{|\frac{1}{\sqrt{\widehat{D}(Y,Y)\widehat{D}(X_j,X_j)}} - \frac{1}{\sqrt{D(Y,Y)D(X_j,X_j)}}| \leq C_d'\sqrt{\frac{t}{n}}\right\},$$

where we let $C_d' = \frac{C_d}{2}(\frac{\widetilde{c}^2}{2})^{-3/2}$.

By (28), we conclude that $\mathcal{A} \subseteq \mathcal{B}$. Therefore, we obtain,

$$\begin{aligned}\mathbb{P}(\mathcal{B}^c) &= \mathbb{P}\left(|\frac{1}{\sqrt{\widehat{D}(Y,Y)\widehat{D}(X_j,X_j)}} - \frac{1}{\sqrt{D(Y,Y)D(X_j,X_j)}}| \geq C_d'\sqrt{\frac{t}{n}}\right) \\ &\leq \mathbb{P}(\mathcal{A}^c) \\ &\leq 56\exp\{-t\}. \end{aligned} \tag{29}$$

By Assumption 1 and 2, we know that both $\frac{1}{\sqrt{D(Y,Y)D(X_j,X_j)}}$ and $D(Y,X_j)$ are upper bounded. Consequently, once again following similar argument to that in (18), with (26), (23) and (29), we

can conclude that there exists a universal constant C_v, such that,

$$\begin{aligned}\mathbb{P}\left(|\hat{\omega}_j^\tau - \omega_j| \geq C_v\sqrt{\frac{t}{n}}\right) &\leq 2 \times (56\exp\{-t\} + 14\exp\{-t\}) \\ &= 140\exp\{-t\}.\end{aligned}$$

By picking $t = C\log p$ with $C > 1$, under Assumption 3, we know that $C_v\sqrt{C} < K$. Therefore, we obtain

$$\mathbb{P}\left(\max_{1\leq j\leq p}|\hat{\omega}_j^\tau - \omega_j| \geq K\sqrt{\frac{\log p}{n}}\right) \leq \mathbb{P}\left(\max_{1\leq j\leq p}|\hat{\omega}_j^\tau - \omega_j| \geq C_v\sqrt{\frac{C\log p}{n}}\right) \leq 140p^{1-C}.$$

Furthermore, note that $\mathcal{D} \nsubseteq \hat{\mathcal{D}}$ indicates that there exists some $j \in \mathcal{D}$, such that $\hat{\omega}_j^\tau \leq K\sqrt{\frac{\log p}{n}}$. Hence, by Assumption 3, we have that $|\hat{\omega}_j^\tau - \omega_j| \geq K\sqrt{\frac{\log p}{n}}$. Consequently, we have

$$\mathcal{P}\left(\mathcal{D} \nsubseteq \hat{\mathcal{D}}\right) \leq \mathcal{P}\left(\exists j \in \mathcal{D} : |\hat{\omega}_j^\tau - \omega_j| \geq K\sqrt{\frac{\log p}{n}}\right) \leq 140sp^{-C}.$$

Therefore, we conclude,

$$\mathcal{P}\left(\mathcal{D} \subseteq \hat{\mathcal{D}}\right) \geq 1 - 140sp^{-C},$$

which completes the proof. □

S4.3 Proof of Theorem 2

Proof. By Proposition 2, the statistics W_j's, $j \in \mathcal{D}^c$ are asymptotically symmetric, and Assumption S4 ensures that the correlations among them are weak. Therefore, Theorem 2 follows from arguments similar to those used in the proof of Proposition 2.2 in Dai et al. (2023). □

S4.4 Proof of Proposition 2

Proof. Note that it suffices to show that, uniformly over all $j \in \mathcal{D}^c$, the estimator $\hat{\omega}_j^\tau$ satisfies the following Berry–Esseen inequality with some limiting distribution Z_j,

$$\max_{j\in\mathcal{D}^c}\sup_{t\in\mathbb{R}} |\mathbb{P}(\sqrt{n}\hat{\omega}_j^\tau \leq t) - \mathbb{P}(Z_j \leq t)| = o(1). \tag{30}$$

The proof then follows by applying an argument similar to that in the proof of Lemma A.1 in Guo et al. (2023).

In the following proof of (30), for convenience, we assume $\omega_j = 0$ for $j \in \mathcal{D}^c$ and take $\delta = 2$ as specified in Assumption S1. We use M, M' to denote universal constants, whose value may vary across different inequalities.

For any $j \in \mathcal{D}^c$, in analogy with kernel $\mathbf{H}$ defined in Assumption S3, we define its truncated counterpart,

$$\mathbf{H}_\tau = \begin{pmatrix} \psi_{\tau_y}(|Y_1 - Y_2|)\psi_{\tau_j}(|X_{1j} - X_{2j}|) - S_1^{*j} \\ \psi_{\tau_y'}(|Y_1 - Y_2|) - S_{2,1}^{*j} \\ \psi_{\tau_j'}(|X_{1j} - X_{2j}|) - S_{2,2}^{*j} \\ \frac{1}{6}\sum_{k\neq\ell\neq m\in\{1,2,3\}} \psi_{\tau_y}(|Y_k - Y_\ell|)\psi_{\tau_j}(|X_{mj} - X_{\ell j}|) - S_3^{*j} \end{pmatrix}$$

Consequently, we define $\mathbf{G}_\tau = \mathbb{E}(\mathbf{H}_\tau|(Y_1, X_{1j}))$ and $\mathbf{V}_\tau = \mathbb{E}(\mathbf{G}\mathbf{G}^\top)$. Following an argument similar to the proof of Lemma S2, one can show that $\mathbb{E}(\|\mathbf{H}_\tau\|_2^3) < \infty$ and $\|\mathbf{V} - \mathbf{V}_\tau\|_\infty \lesssim \log p/n$, where $\|\cdot\|_\infty$ denotes the maximum absolute entry of a matrix. Consequently, by Assumption S3, we can conclude that the largest and smallest eigenvalues of $\mathbf{V}_\tau$ are also bounded away from 0 and above by some constant.

Let $\mathbf{R} = \begin{pmatrix} 2 & & & \\ & 2 & & \\ & & 2 & \\ & & & 3 \end{pmatrix}$, where the diagonal entries correspond to the order of the kernel $\mathbf{H}_\tau$.

Define $\mathbf{a} = \frac{1}{D(Y,Y)D(X_j,X_j)}(1, S_{2,2}^j, S_{2,1}^j, -2)^\top$, and set $\sigma_{j\tau}^2 = \mathbf{a}^\top\mathbf{R}\mathbf{V}_\tau\mathbf{R}\mathbf{a}$, $\sigma_j^2 = \mathbf{a}^\top\mathbf{R}\mathbf{V}\mathbf{R}\mathbf{a}$. Since the eigenvalues of both $\mathbf{V}$ and $\mathbf{V}_\tau$ are bounded, and by Assumption 2, $D(Y, Y)$ and $D(X_j, X_j)$ are bounded away from 0, it follows that both σ_j and $\sigma_{j\tau}$ are bounded.

We define

$$\widetilde{\omega}_j^{\tau} = \frac{(\widehat{S}_1^{\tau_j,\tau_y} - S_1^{*j}) + (\widehat{S}_{2,1}^{\tau_y'} - S_{2,1}^{*j})S_{2,2}^{*j} + (\widehat{S}_{2,2}^{\tau_j'} - S_{2,2}^{*j})S_{2,1}^{*j} - 2(\widehat{S}_3^{\tau_j,\tau_y} - S_3^{*j})}{D(Y,Y)D(X_j,X_j)}.$$

Note that by Assumption S1, we have $\mathbb{E}(\|\mathbf{H}_\tau\|_2^3), \mathbb{E}(\|\mathbf{H}\|_2^3) < \infty$. Combined with Assumption S3, this allows us to apply Lemma S3, from which we obtain that

$$\sup_t \left|\mathbb{P}(\sqrt{n}\widetilde{\omega}_j^{\tau} \le t) - \Phi(t/\sigma_{j\tau})\right| \lesssim 1/\sqrt{n}, \tag{31}$$

where $\Phi(\cdot)$ is the CDF of standard normal. Note that in Lemma S3, all kernels are assumed to be of order 2. However, after examining the relevant proofs in Götze (1987), similar results can be extended to kernels of other orders.

Observe that

$$\begin{aligned}
\sqrt{n}\widehat{\omega}_j^{\tau} &= \sqrt{n}\frac{\widehat{S}_1^{\tau_j,\tau_y} + \widehat{S}_{2,1}^{\tau_y'}\widehat{S}_{2,2}^{\tau_j'} - 2\widehat{S}_3^{\tau_j,\tau_y}}{\widehat{D}(Y,Y)\widehat{D}(X_j,X_j)} \\
&= \sqrt{n}\frac{D(Y,Y)D(X_j,X_j)}{\widehat{D}(Y,Y)\widehat{D}(X_j,X_j)}\left[\widetilde{\omega}_j^{\tau} + \frac{S_1^{*j} + S_{2,1}^{*j}S_{2,2}^{*j} - 2S_3^{*j} + (\widehat{S}_{2,1}^{\tau_y'} - S_{2,1}^{*j})(\widehat{S}_{2,2}^{\tau_j'} - S_{2,2}^{*j})}{D(Y,Y)D(X_j,X_j)}\right] \\
&= (\sqrt{n}\widetilde{\omega}_j^{\tau} + \widehat{b})\frac{1}{1+\widehat{r}},
\end{aligned}$$

where we have $\widehat{r} = \frac{\widehat{D}(Y,Y)\widehat{D}(X_j,X_j)}{D(Y,Y)D(X_j,X_j)} - 1$, and $\widehat{b} = \sqrt{n}\frac{S_1^{*j}+S_{2,1}^{*j}S_{2,2}^{*j}-2S_3^{*j}+(\widehat{S}_{2,1}^{\tau_y'}-S_{2,1}^{*j})(\widehat{S}_{2,2}^{\tau_j'}-S_{2,2}^{*j})}{D(Y,Y)D(X_j,X_j)}$.

It follows by (27), that with probability at least $1 - 56/p^C$, we have that

$$|\widehat{D}(Y,Y)\widehat{D}(X_j,X_j) - D(Y,Y)D(X_j,X_j)| \le M\sqrt{\log p/n},$$

where M is some universal constant, and C is the constant specified in Theorem 1. Hence, by Assumption 2 that $D(Y,Y)D(X_j,X_j)$ is bounded away from 0, we can then conclude that

$$|\widehat{r}| \lesssim \sqrt{\log p/n}. \tag{32}$$

To show that the bias term $\widehat{b}$ is also negligible, note that by Lemma S2, we have

$$\sqrt{n}(S_1^{*j} + S_{2,1}^{*j} S_{2,2}^{*j} - 2S_3^{*j}) \lesssim \log p/\sqrt{n}. \tag{33}$$

Furthermore, from (17), we have

$$\mathbb{P}\left(|\widehat{S}_{2,2}^{\tau_j'} - S_{2,2}^{j}| \geq C_{2,2}\sqrt{\frac{\log p}{n}}\right) \leq 2/p^C, \tag{34}$$

where again C is the constant from Theorem 1 and $C_{2,2}$ is a constant appearing in (17).

Applying Lemma S3 to the second kernel in $\mathbf{H}_\tau$, we can get that for some constant M

$$\mathbb{P}\left(\sqrt{n}\left|\widehat{S}_{2,1}^{\tau_y'} - S_{2,1}^{*j}\right| \leq n^{1/4}\right) \geq 2\Phi\left(\frac{n^{1/4}}{2\sqrt{[\mathbf{V}_\tau]_{22}}}\right) - 2M/\sqrt{n}. \tag{35}$$

Note that

$$\begin{aligned}&\left|\Phi(t/\sqrt{[\mathbf{V}_\tau]_{22}}) - \Phi(t/\sqrt{[\mathbf{V}]_{22}})\right| \\ &= \frac{1}{\sqrt{2\pi}}\exp\{-z_t^2/2\}t\left|\frac{[\mathbf{V}]_{22} - [\mathbf{V}_\tau]_{22}}{\sqrt{[\mathbf{V}]_{22}[\mathbf{V}_\tau]_{22}}(\sqrt{[\mathbf{V}]_{22}} + \sqrt{[\mathbf{V}_\tau]_{22}})}\right|,\end{aligned} \tag{36}$$

for some z_t between $t/\sqrt{[\mathbf{V}_\tau]_{22}}$ and $t/\sqrt{[\mathbf{V}]_{22}}$. Since $\sqrt{[\mathbf{V}_\tau]_{22}}$ and $\sqrt{[\mathbf{V}]_{22}}$ are both bounded, we can conclude that $\sup_{t\in\mathbb{R}}\exp\{-z_t^2/2\}t$ is bounded. Therefore, we have for some constant M

$$\sup_t\left|\Phi(t/\sqrt{[\mathbf{V}_\tau]_{22}}) - \Phi(t/\sqrt{[\mathbf{V}]_{22}})\right| \leq M\log p/n. \tag{37}$$

Combining (33), (34), (35) and (37), we conclude that with probability at least $2(\Phi\left(\frac{n^{1/4}}{2\sqrt{[\mathbf{V}]_{22}}}\right) - M/\sqrt{n} - M\log p/n - 1/p^C)$, we have

$$|\widetilde{b}| \lesssim (\log p)^{1/2}/(n)^{1/4}. \tag{38}$$

Define $\mathcal{B} = \left\{|\widehat{r}| \leq M'\sqrt{\log p/n}|\widehat{b}| \leq M'(\log p)^{1/2}/(n)^{1/4},\right\}$. Then it follows from (32) and (38)

that $\mathbb{P}(\mathcal{B}) \to 1$ as $(n,p) \to \infty$. Note that

$$
\begin{aligned}
&\mathbb{P}(\sqrt{n}\widehat{\omega}_j^\tau \le t) - \Phi(t/\sigma_j) \\
&= \mathbb{P}(\sqrt{n}\widetilde{\omega}_j^\tau \le t - \widehat{b} + t\widehat{r}) - \Phi(t/\sigma_j) \\
&\le \mathbb{P}\left(\sqrt{n}\widetilde{\omega}_j^\tau \le t + M'(\log p)^{1/2}/(n)^{1/4} + tM'\sqrt{\log p/n}\right) - \Phi(t/\sigma_j) + \mathbb{P}(\mathcal{B}^c) \\
&\le \mathbb{P}\left(\sqrt{n}\widetilde{\omega}_j^\tau \le t + M'(\log p)^{1/2}/(n)^{1/4} + tM'\sqrt{\log p/n}\right) - \Phi\left(\frac{t + M'(\log p)^{1/2}/(n)^{1/4} + tM'\sqrt{\log p/n}}{\sigma_{j\tau}}\right) \quad (39) \\
&+ \Phi\left(\frac{t + M'(\log p)^{1/2}/(n)^{1/4} + tM'\sqrt{\log p/n}}{\sigma_{j\tau}}\right) - \Phi(t/\sigma_j) \quad (40) \\
&+ \mathbb{P}(\mathcal{B}^c).
\end{aligned}
$$

By (31), we conclude that (39) converges to 0 uniformly over all t. Following the argument used in (36), we obtain that (40) also converges to 0 uniformly over all t. An analogous argument yields the reverse inequality, and thus we conclude that

$$
\max_{j \in \mathcal{D}^c} \sup_t |\mathbb{P}(\sqrt{n}\widehat{\omega}_j^\tau \le t) - \Phi(t/\sigma_j)| = o(1),
$$

which completes the proof. □

S4.5 Proof of Lemma S2

Proof. Note that for $|Y - \widetilde{Y}| \ge \tau_y$, we have that

$$
\begin{aligned}
\frac{|Y - \widetilde{Y}| - \tau_y}{|Y - \widetilde{Y}|} \bigg/ \frac{|Y - \widetilde{Y}|^{\delta+2}}{\tau_y^{\delta+2}} &= \frac{\tau_y^{\delta+2}(|Y - \widetilde{Y}| - \tau_y)}{|Y - \widetilde{Y}|^{\delta+3}} \\
&\le \max_{r \ge 1} \frac{r-1}{r^{\delta+3}} \\
&= \frac{(\delta+2)^{\delta+2}}{(\delta+3)^{\delta+3}}.
\end{aligned}
$$

Then, following a similar argument in (3), we can show that $S_1^j - S_1^{*j} \lesssim (\log p/n)^{(\delta+2)/4}$. We complete the proof by applying similar arguments to $S_2^j - S_2^{*j}$ and $S_3^j - S_3^{*j}$. □

References

Arcones, M. A. (1995), "A Bernstein-type inequality for U-statistics and U-processes," *Statistics & Probability Letters*, 22, 239–247.

Dai, C., Lin, B., Xing, X., and Liu, J. S. (2023), "False discovery rate control via data splitting," *Journal of the American Statistical Association*, 118, 2503–2520.

Fan, J., Li, Q., and Wang, Y. (2017), "Estimation of high dimensional mean regression in the absence of symmetry and light tail assumptions," *Journal of the Royal Statistical Society: Series B (Statistical Methodology)*, 79, 247–265.

Fan, J. and Lv, J. (2008), "Sure independence screening for ultrahigh dimensional feature space," *Journal of the Royal Statistical Society: Series B (Statistical Methodology)*, 70, 849–911.

Götze, F. (1987), "Approximations for multivariate U-statistics," *Journal of Multivariate Analysis*, 22, 212–229.

Guo, X., Ren, H., Zou, C., and Li, R. (2023), "Threshold selection in feature screening for error rate control," *Journal of the American Statistical Association*, 118, 1773–1785.

He, D., Zhou, Y., and Zou, H. (2021), "On sure screening with multiple responses," *Statistica Sinica*, 31, 1749–1777.

He, K., Kang, J., Hong, H. G., Zhu, J., Li, Y., Lin, H., Xu, H., and Li, Y. (2019), "Covariance-insured screening," *Computational Statistics & Data Analysis*, 132, 100–114.

Hoeffding, W. (1963), "Probability inequalities for sums of bounded random variables," *Journal of the American Statistical Association*, 58, 13–30.

Ke, H., Ren, Z., Qi, J., Chen, S., Tseng, G. C., Ye, Z., and Ma, T. (2022), "High-dimension to high-dimension screening for detecting genome-wide epigenetic and noncoding RNA regulators of gene expression," *Bioinformatics*, 38, 4078–4087.

Li, R., Zhong, W., and Zhu, L. (2012), "Feature screening via distance correlation learning," *Journal of the American Statistical Association*, 107, 1129–1139.
